# Therapeutic potential of exosomes in periodontal regeneration: Immunomodulatory and tissue-repair mechanisms

**DOI:** 10.3389/fimmu.2025.1675707

**Published:** 2025-11-28

**Authors:** Shuang Yang, Chunyu Han, Qihui Wang, Yicen Ai, Dezhou Wang, Wenzhi Song

**Affiliations:** Department of Stomatology, China-Japan Union Hospital, Jilin University, Changchun, China

**Keywords:** periodontitis, exosomes, immunomodulation, immune cells, periodontal tissue regeneration, alveolar bone regeneration

## Abstract

Periodontitis, a chronic inflammatory disease leading to irreversible tissue destruction, is a highly prevalent oral disease. The clinical management of periodontitis is challenging because conventional treatments like mechanical debridement and antibiotic therapy lack sufficient regenerative efficacy to achieve functional periodontal restoration. In recent years, exosomes have received widespread attention as cell-free therapeutic agents for periodontal tissue regeneration. This article reviews the dual role of exosomes in modulating immune response and promoting tissue repair, and briefly describes the exosome delivery systems studied so far. The aim of this review is to emphasize the important position occupied by cell-derived exosomes in the treatment of periodontitis as well as the main mechanisms, and to explore novel targets for the treatment of periodontitis.

## 1 Introduction

**Figure 1:**
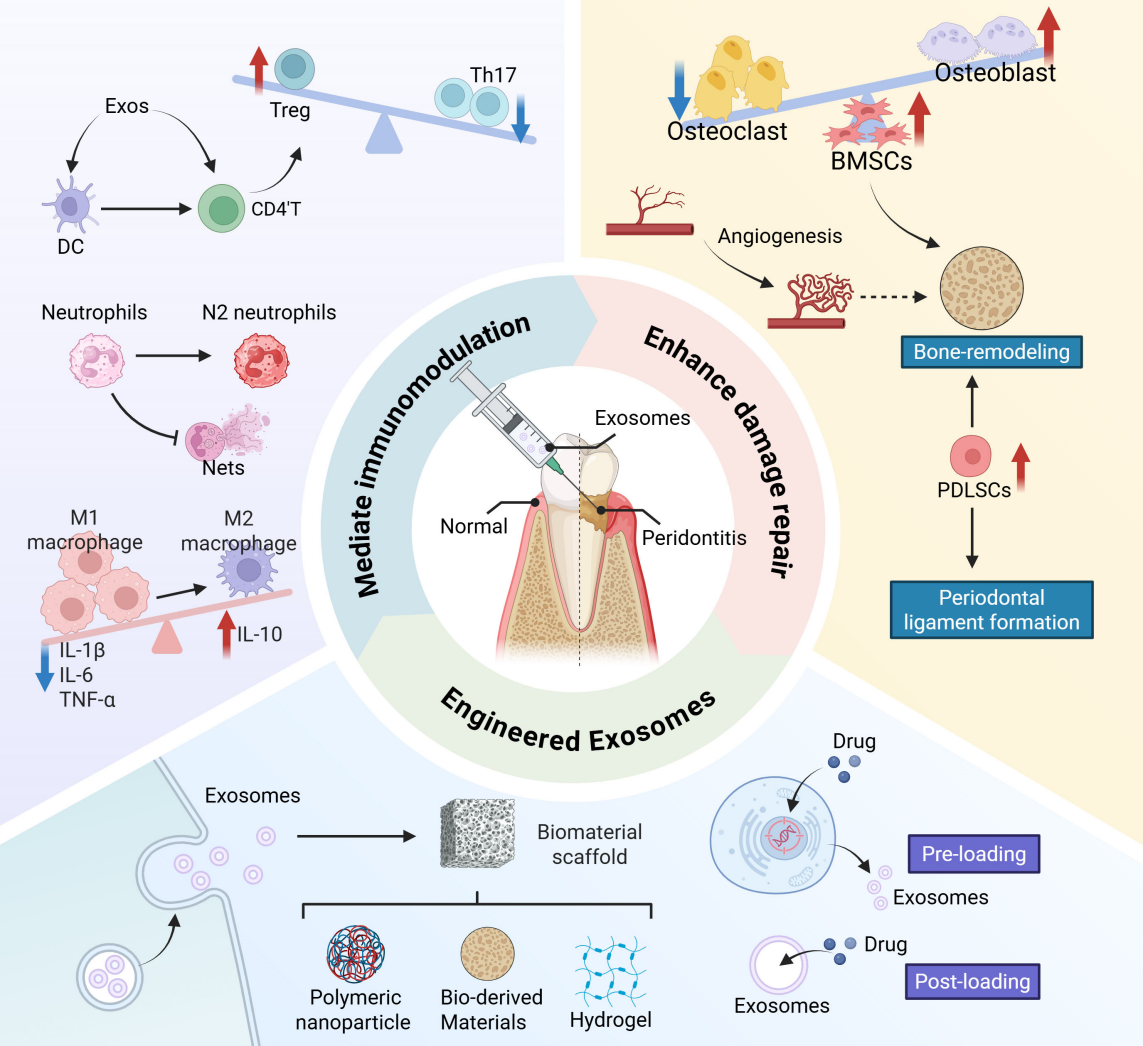
Schematic diagram of exosomes improving inflammatory microenvironment and promoting periodontal regeneration.

Periodontitis is a major global chronic inflammatory and destructive condition, the major cause of tooth loss worldwide (1–3) and a potential risk factor for a variety of systemic diseases. A large amount of evidence supports the association between periodontal disease and various systemic diseases, including cardiovascular diseases, rheumatoid arthritis and diabetes (4–6). For example, the risk of suffering an acute myocardial infarction has been shown to be two to four times higher in patients with moderate to severe periodontitis (7). Periodontitis is characterized by pathologic loss of periodontal attachment and progressive alveolar bone resorption (8). The ultimate goal of periodontal treatment is to control the infections and reconstruct the structure and function of periodontal tissues including cementum, alveolar bone, periodontal ligament (PDL) and gingival connective tissue. The routine treatment for periodontal disease mainly includes basic therapy, guided tissue regeneration (GTR), and guided bone regeneration (GBR). However, these approaches are associated with significant limitations. Non-surgical treatments, such as long-term antibiotic use, carry the risk of inducing bacterial resistance, while mechanical debridement alone fails to stimulate regeneration of lost bone tissue. Surgical interventions, including GTR and GBR, are constrained by inherent surgical risks, strict indication criteria, and technical challenges such as membrane exposure and inadequate vascularization (2, 9, 10). Consequently, current therapeutic strategies for periodontitis primarily aim to control infection but often fall short of reliably reconstructing the supporting structures and biological connections of periodontal tissues damaged by the disease. As a result, achieving predictable periodontal regeneration remains a major clinical challenge (11–13). Therefore, the current research trend has shifted towards developing tissue engineering and cell-based techniques for periodontal regeneration (14). Mesenchymal stem cell (MSC) therapy has been shown to have regenerative potential in the treatment of periodontal defects. However, the clinical application of exogenous cell therapy faces several challenges, including the requirement for large-scale cell expansion and specialized technical expertise, which substantially elevates treatment costs. Moreover, the efficacy of transplanted exogenous stem cells is highly dependent on donor-specific factors and the pathophysiological microenvironment of the recipient’s lesion (15). Additionally, cell-based therapies are associated with inherent risks such as immunogenic responses, potential disease transmission, limited cell survival, and tumorigenicity (16). Consequently, the therapeutic benefits of MSCs are increasingly attributed not to their direct differentiation capacity but rather to their paracrine functions, particularly mediated through the secretion of exosomes or small extracellular vesicles (17). Given these considerations, this review aims to synthesize current understanding of the therapeutic potential of exosomes in periodontal regeneration, emphasizing their dual roles in immunomodulation and tissue repair. This review comprehensively elucidates the cellular and molecular regulatory effects of exosomes on periodontitis-associated immune cells (such as neutrophils, macrophages, and T cells) and regenerative tissues (including gingival epithelium, alveolar bone, and periodontal ligament), providing robust evidence for the role of exosomes in promoting comprehensive periodontal regeneration. Furthermore, this review outlines recent advances in engineered exosome delivery systems and provides a forward-looking analysis of related clinical translation challenges and future research directions (**Figure 1**).

## 2 Exosomes

**Figure 2:**
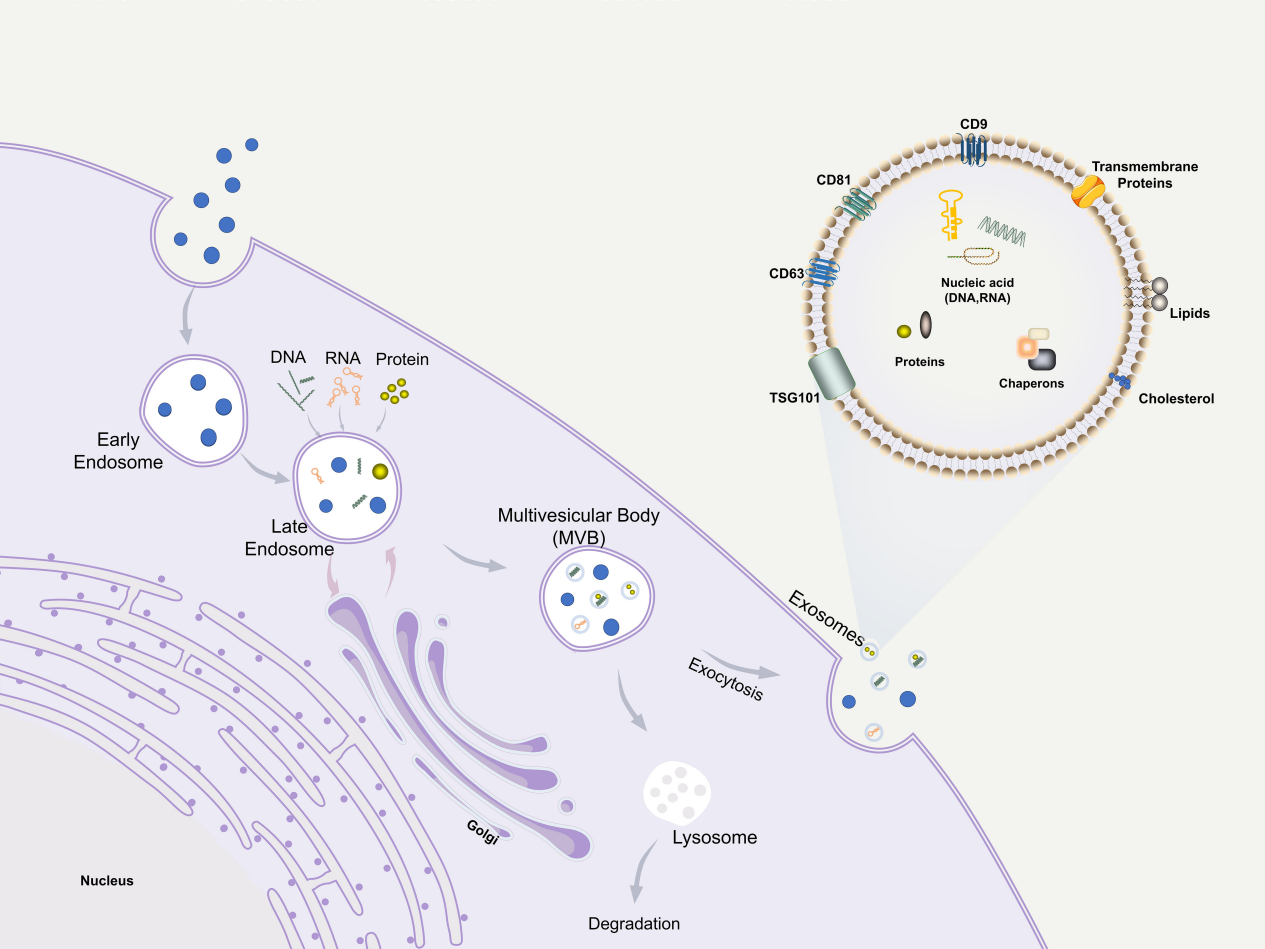
Biogenesis, structure, and function of exosomes.

Extracellular vesicles (EVs) are membrane-bound particles released by cells into the extracellular environment, which are broadly classified into three major subtypes based on their biogenesis and size: exosomes, microvesicles, and apoptotic bodies (18, 19). Exosomes, the smallest among these EVs, range from 30 to 150 nm in diameter and possess a lipid bilayer structure. They are secreted by a wide variety of cell types and play a crucial role in intercellular communication by transferring bioactive molecules—such as proteins, lipids, and genetic materials including mRNA, miRNA, and snRNA—from donor to recipient cells (19–22). In terms of biogenesis, apoptotic bodies (800–5000 nm) and microvesicles (200–1000 nm) are generated through direct budding from the plasma membrane (23). In contrast, exosomes are derived from the endosomal pathway: they are formed as intraluminal vesicles via inward budding of the endosomal membrane within multivesicular bodies (MVBs) and are subsequently released into the extracellular space upon fusion of MVBs with the plasma membrane (24) (**Figure 2**). This distinct mode of formation underscores the unique molecular and functional identity of exosomes among EVs. Exosomes can directly fuse with the receptor cytoplasmic membrane and deliver information, being internalized through endocytosis or phagocytosis (25). Exosomes as important mediators of paracrine effects retain almost all advantages of source cells. In addition to their unique advantages of being cell-free, ready-to-use, easy to store, and easy to reformulate to support different routes of administration (26), exos have lower immunogenicity and better biocompatibility than cell-based therapies, resulting in lower post-transplantation immune-related adverse reactions (27–29). Moreover, the composition of the contents of exosomes from different types of cells is different, and even for cells of the same type, the exos they secrete are highly heterogeneous because of the different environments in which they are located (28). This heterogeneity, while presenting a challenge for standardization, also offers opportunities for sourcing exosomes with specific desired functions. Recent studies have shown that exosomes are effective in various animal models of tissue damage and have emerged as potential therapies for inflammatory diseases and tissue injuries (21). The potential of exosomes in periodontal regeneration has been demonstrated in recent years.

## 3 Modulating the inflammatory and immune response

Extensive research on cellular and animal models has begun to unravel the complex immunopathogenesis of periodontitis. Recent studies have shown that the progression of periodontitis is an organized cellular response, with tissue-resident dendritic cells (DCs), B cells, plasma cells, macrophage, T cells. Neutrophils respond early and rapidly to subgingival pathogens, producing associated enzymes to kill the pathogens and releasing cytokines and chemokines, which attract immune cells such as macrophages, T cells, and DCs to the site, participating in the periodontal immune response. This section will explore the effects of exosomes on the modulation of immune responses in various immune cells within the periodontal tissues.

### 3.1 Neutrophils

Neutrophils are polymorphonuclear leukocytes, the most prevalent immune cells in the human body, constituting 60-70% of all circulating leukocytes, and the proportion of neutrophils in the oral mucosa is significantly higher than that in other barrier tissues (30). Neutrophils are the first line of defense of the innate immune system against pathogens (31) and the balance between their activity and apoptosis plays a crucial role in maintaining periodontal homeostasis. In periodontitis, this balance is disrupted; overactive neutrophils triggers excessive release of proteolytic enzymes, reactive oxygen species (ROS), and neutrophil extracellular traps (NETs). Recent clinical evidence has demonstrated that elevated levels of NETs (32) and ROS (33) in gingival tissues and gingival crevicular fluid of periodontitis patients are positively correlated with the severity of periodontitis and the degree of tissue destruction. Studies have indicated that NETs not only contribute directly to tissue damage, but may also perpetuate inflammation by promoting cytokine release and activating other immune pathways (34). This pathological accumulation of cytotoxic mediators creates a self-perpetuating cycle of inflammation that drives progressive destruction of periodontal structures (35).

The therapeutic potential of exosomes lies in their ability to recalibrate this dysregulated neutrophil response. Studies found that MSCs-exos could reduce tissue damage by inhibiting NETs, increasing anti-inflammatory cytokines, and reducing immune responses (36, 37) Exosomes can suppress the destructive NETosis process, as mechanistically demonstrated by Morishima et al., who identified exosomal miR-125a-3p as a direct mediator of this inhibition (38). Furthermore, they can induce neutrophil apoptosis, thereby facilitating the clearance of these cells, shifting macrophages toward an anti-inflammatory phenotype, and accelerating inflammation resolution (39). Another facet of their function is the induction of N2 polarization in neutrophils, which promotes the release of pro-angiogenic factors like BV8, thereby stimulating angiogenesis and tissue repair (40). Conversely, other studies present a seemingly paradoxical effect: exosomes may prolong neutrophil lifespan (41) and enhance phagocytosis via anti-apoptotic miRNAs (e.g., let-7 family, IL-6 mRNA) (42). This evidence raises a critical question: do exosomes primarily eliminate overactive neutrophils or reprogram them to enhance their phagocytic function?

This apparent contradiction likely underscores the context-dependent duality of exosome therapy, rather than representing mutually exclusive actions. The effect on neutrophils is probably determined by variables such as the specific exosomal cargo (which is influenced by the cellular source and preconditioning), the local inflammatory microenvironment, and the temporal stage of the disease. This duality demonstrates the strategic potential of applying exosomes to achieve tailored therapeutic outcomes—by eliminating pathological neutrophils or reprogramming their reparative functions during different stages of inflammation. However, translating this promise into reality faces significant hurdles. Therefore, a central consideration for future therapeutic development will be striking a precise balance between suppressing detrimental neutrophil functions and preserving or enhancing their beneficial roles.

### 3.2 Macrophages

As an important component of innate immunity, macrophages exhibit remarkable diversity and plasticity, playing critical roles in inflammatory responses. Functionally, within local tissue microenvironments, macrophages are broadly categorized into classically activated (M1), alternatively activated (M2), and unactivated (M0) states (43, 44). M1 macrophages are induced by Th1 cytokines like Interferon-γ (IFN-γ) or microbial products like lipopolysaccharide (LPS), and mediate pro-inflammatory responses through the production of cytokines such as IL-1β, Tumor necrosis factor-alpha (TNF-α), and IL-6. In contrast, M2 macrophages are induced by cytokines IL-4 and IL-13, characterized by their secretion of anti-inflammatory mediators, including IL-10, Transforming growth factor beta (TGF-β), and Vascular endothelial growth factor (VEGF) (45).

In periodontal tissues, macrophages play a key role in mobilizing host defense against microbial infection and maintaining tissue homeostasis. However, excessive activation towards the M1 phenotype can lead to periodontal tissue destruction and aggravate periodontitis (46, 47). Therefore, a timely and appropriate phenotypic transition from pro-inflammatory (M1) to anti-inflammatory (M2) macrophages is critical for resolving inflammation and treating periodontitis (48).

Through RNA sequencing and gene ontology analysis, Yue et al. found that the most significant biological activities of macrophages affected by exosome treatment were metabolic processes and cell differentiation (49). MSC-derived exosomes can significantly alter macrophage M1 to M2 phenotypes (50). Current studies have shown that Gingival mesenchymal stem cells-derived exosomes (GMSCs-exos) (25), human periodontal ligament cells-derived exosomes (hPDLCs-exos) (51), dental pulp stem cells-derived exosome (DPSC-exos) (52), M2 macrophage-derived exosome (M2-exos) (48), and adipose mesenchymal stem cells-derived exosomes (ADSC-exos) (53) can positively promote the conversion of M1 macrophages to M2 macrophages, reduce the production of pro-inflammatory factors and stimulate the production of anti-inflammatory cytokines, inhibiting periodontal bone loss and treating periodontitis. Moreover, it has been demonstrated that macrophage-derived exosomes can effectively clear MRSA/E. coli (54).

**Figure 3:**
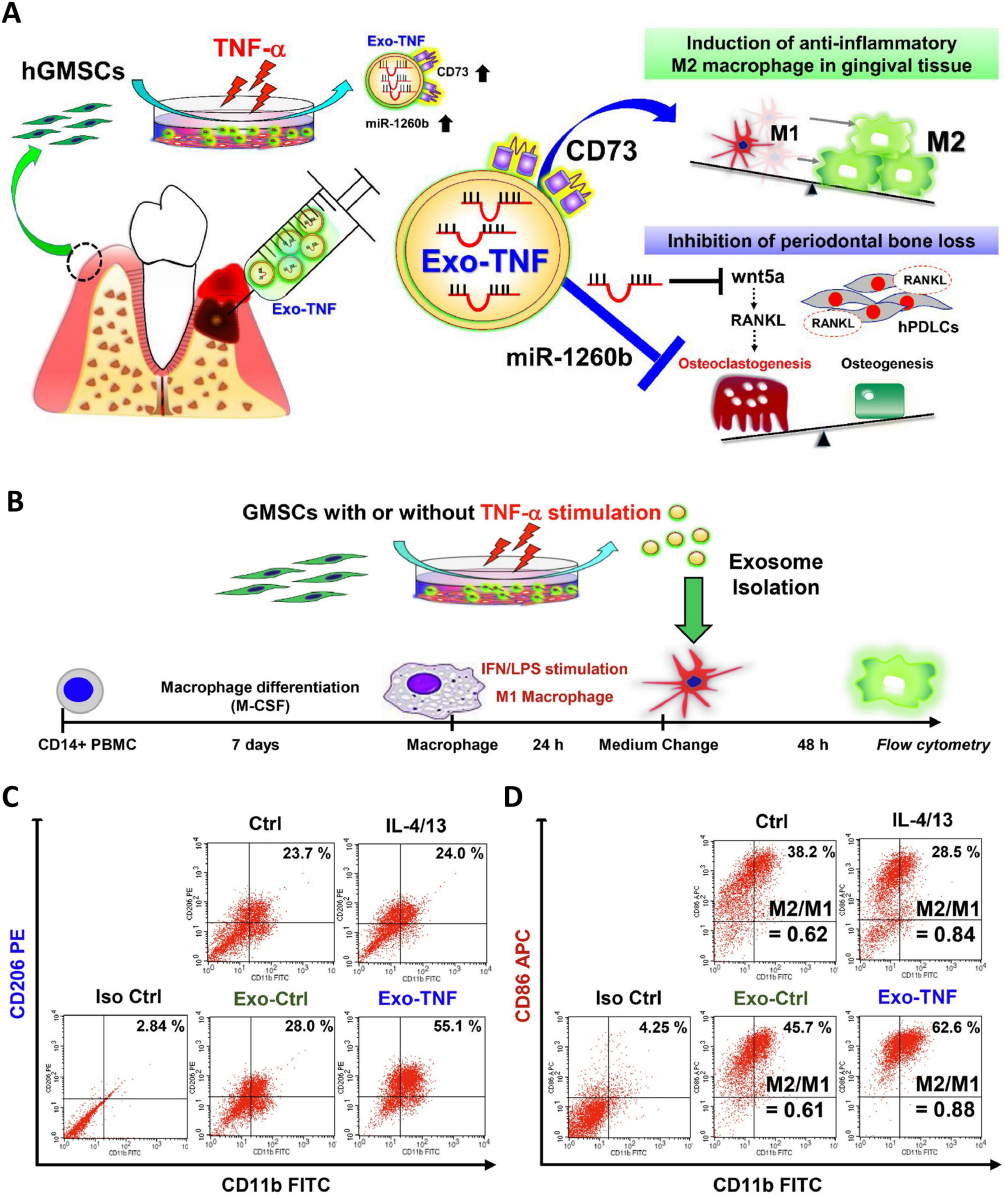
Effect of TNF-α-treated GMSC-derived exosomes on macrophage polarization. **(A)** Mechanism diagram of Exo-TNF treatment of periodontitis. **(B)** Strategy diagram to validate the capacity of exosomes to promote M1 to M2 macrophages. **(C)** Effects exosomes on CD86 expression in M1 macrophages. The percentage of double-positive cells (CD11b+ CD206+) was analyzed to represent the ratio of M2 macrophages. **(D)** The percentage of double positive cells (CD11b+ CD86+) was analyzed to represent M1 macrophages. M2/M1 balance was expressed by the ratio of (CD11b+ CD206+)/(CD11b+ CD86+) macrophage populations (51). ^©^ 2020 Acta Materialia Inc.

Several studies have shown that exosomes contain miRNAs such as miR-223 (53), miR-1246 (52), miR-21-5p (55) and miR-182 (56), which play important roles in regulating macrophage phenotypes. This regulation may be mediated through multiple signaling pathways: Zhang et al. identified Toll-like receptor 4 (TLR4) suppression by exosomes as a key driver (56); miR-23a-3p in MSCs-exos inhibits IRF1 expression and the NF-κB signaling pathway to promote M2 phenotypes; Exo-181b was shown to significantly down-regulate PRKCD expression, thereby enhancing p-AKT, polarizing macrophages to M2 (57); And ADSCs-exos regulate macrophages via miR-451a/MIF (58). Additionally, Cmklr1 (ChemR23), the receptor for Resolvin E1, was significantly increased in macrophages by exosomes, which is conducive to the suppression of periodontal tissue inflammation and the reduction of bone loss (49). Furthermore, pretreatment of cells enhances this immunomodulatory capacity of exosomes. Wang et al. proposed that under conditions of circulating tensile stress, PDLCs-exos can Suppress IL-1β Production through the Inhibition of the NF-κB Signaling Pathway in LPS-stimulated Macrophages, which contribute to the maintenance of periodontal immune/inflammatory homeostasis (59). Nakao et al. demonstrated that Exosomes from TNF-α-treated human gingiva-derived MSCs(Exo-TNF) have a greater ability to convert macrophage phenotype from M1 to M2 by examining the expression of M1 (CD86) and M2 markers (CD206), and it was further demonstrated that high CD73 expression of Exo-TNF is important for M2 macrophage polarization (**Figure 3**) (51).

However, the functional consequences of exosome-induced M2 polarization are not uniformly straightforward. While M2 macrophages are typically associated with the release of regenerative factors such as Bone Morphogenetic Protein type 2 (BMP-2) and VEGF, which promote osteogenesis (60), studies have shown that the M2 phenotype can also elevate the expression of markers for osteoclastogenesis and enhance osteoclast differentiation (61–63). Exosomes have been found to promote this pro-osteoclastogenic effect (64), thereby presenting a more complex picture. This apparent paradox—whereby the M2 polarization that aids in resolving inflammation may also inadvertently facilitate bone resorption—highlights the insufficiency of the simplistic M1/M2 dichotomy in capturing the functional heterogeneity of macrophage subsets in periodontal healing. The therapeutic outcome is likely determined by the specific M2 subpopulation induced, the timing of polarization, and the integrated signals from the surrounding microenvironment. Consequently, achieving precise induction and control over these complex macrophage responses emerges as a central challenge in developing exosome-based therapies for periodontitis.

### 3.3 T cells

**Figure 4:**
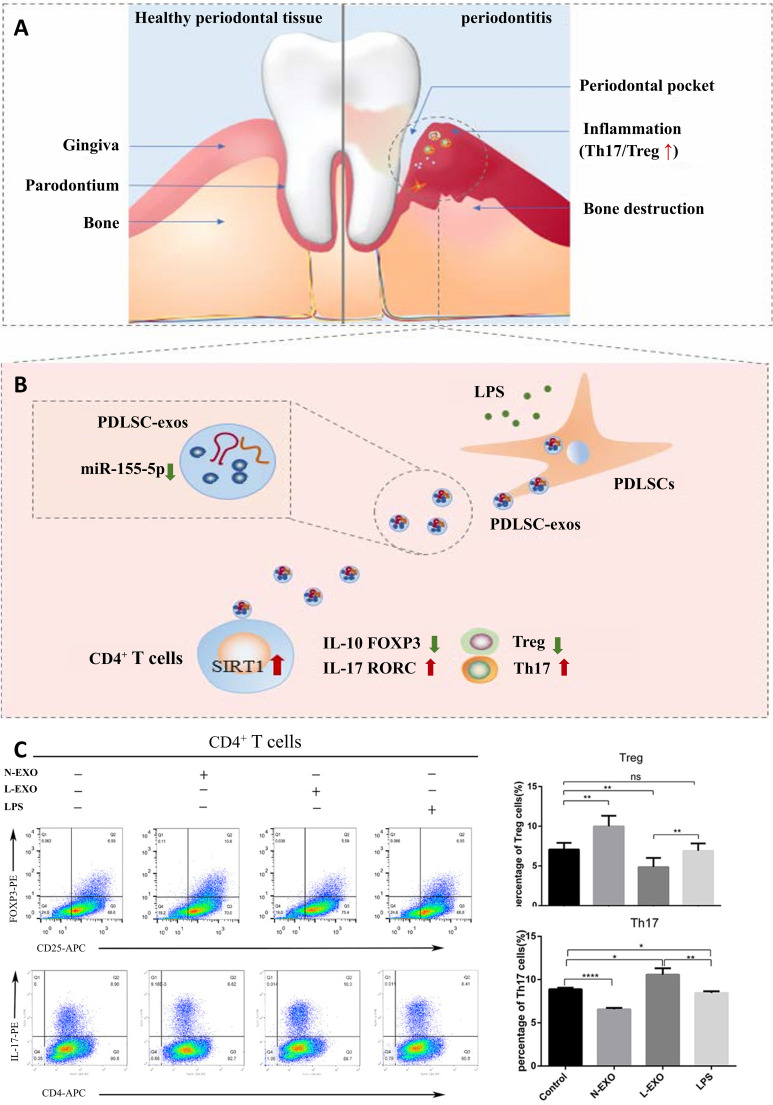
PDLSC-exos improves Th17/Treg balance in periodontitis. **(A)** Th17/Treg imbalance in periodontal tissues. **(B)** PDLSC-exos ameliorate the inflammatory microenvironment through the Th17/Treg/miR-155-5p/SIRT1 regulatory network. **(C)** After 72hr of exosome treatment of CD4+ T cells, the amount of Th17 (CD4+IL-17+) and Treg (CD4+CD25+FOXP3+) was detected by flow cytometry. ns, no significant difference; *p < 0.05; **p < 0.01; ****p<0.0001. N-EXO, exosomes from the normal PDLSCs; L-EXO, exosomes from the PDLSCs stimulated with 1 ug/ml LPS (66). *^©^ 2019 Wiley Periodicals, Inc.*

Within CD4^+^ T lymphocyte populations, the balance between T helper 17 (Th17) and regulatory T (Treg) cells critically influences periodontitis pathogenesis (65). It was found that Th17 were up-regulated or Treg were down-regulated respectively in peripheral blood and periodontal tissues of patients with periodontitis (66). Emerging evidence further positions Tregs as central mediators of tissue repair, suppressing the inflammatory response by producing anti‐inflammatory cytokines such as IL‐10, TGF‐β, and IL‐35 (67, 68), with their local presence demonstrably attenuating experimental periodontitis severity and limiting alveolar bone destruction (69–71). Zheng et al. demonstrated that Periodontal ligament stem cells (PDLSC)-exosomes can be taken up by CD4+ T cells, which in turn regulate Th17/Treg balance (**Figure 4**) (66). In addition, this immune imbalance is orchestrated by DCs, the principal conductors of T cell differentiation (72). At oral lymphoid foci, DCs direct naïve T cell fate determination, selectively promoting either pro-inflammatory Th17 or reparative Treg dominance (73).

Dendritic cell (DC)-derived exosomes demonstrate targeted immunomodulatory effects in inflammatory bone loss contexts (74), with regulatory DC exosomes exhibiting enhanced retention within inflamed gingival tissues (73). These exosomes concurrently impair recipient DC maturation, suppress Th17 effector induction, and promote Treg recruitment, ultimately attenuating alveolar bone destruction by reducing bone-resorptive cytokines (73, 74). This regulatory network is amplified through reciprocal exosomal crosstalk: Treg-derived exosomes reprogram DCs toward anti-inflammatory phenotypes (characterized by increasing IL-10/decreasing IL-6 production) (75), while delivering miRNAs (e.g., Let-7d) that inhibit Th1 proliferation and IFN-γ secretion *in vitro* and *in vivo* (76). Aiello et al. found that Engineered dnIKK2-Treg-EVs further enhance immunomodulation by suppressing IFN-γ^+^ T cells and inducing IL-10 expression (75). Crucially, exosomal miRNAs orchestrate Th17/Treg rebalancing through distinct pathways—PDLSC-derived miR-155-5p targets Sirtuin-1 to suppress Th17 differentiation while expanding Treg populations (66), and 3D-cultured MSC-exosomes restore immune equilibrium in periodontitis via the miR-1246/NFAT5 axis (77). Complementing these mechanisms, PD-L1-engineered HUVEC-exosomes bind PD-1 on T cells to suppress activation and promote osteogenesis (78), collectively establishing exosomes as multifaceted immunotherapeutic agents.

## 4 Repairing and regenerating periodontal tissues

### 4.1 Gingival epithelial barrier regeneration

The gingival epithelium serves as the primary barrier against pathogenic microbial invasion into periodontal tissues, functioning as both a physical shield preventing microbial components from penetrating deeper connective tissues and a key component of nonspecific immunity in periodontal inflammation (79). Compromised integrity of this epithelial barrier is strongly associated with the pathogenesis of periodontal diseases (80). Therapeutic strategies targeting epithelial barrier restoration and immunomodulation have demonstrated efficacy in preventing or ameliorating periodontitis in preclinical studies (81–84).

In rat models of experimental periodontitis, DPSC-exos significantly promote gingival epithelial healing by suppressing the IL-6/JAK2/STAT3 signaling pathway, thereby reducing inflammatory cell infiltration, mitigating tissue damage, and attenuating periodontal inflammation (85). Similarly, ADSC-exos enhance gingival repair by improving human gingival fibroblast migration and collagen synthesis through IL-1RA-mediated anti-inflammatory actions (86, 87). Despite these promising findings, research on exosome-mediated mechanisms within the gingival epithelium remains limited, and their precise regulatory functions require further elucidation. This represents a significant gap in the literature, as effective barrier restoration is fundamental to treating periodontitis.

### 4.2 Bone regeneration

**Figure 5:**
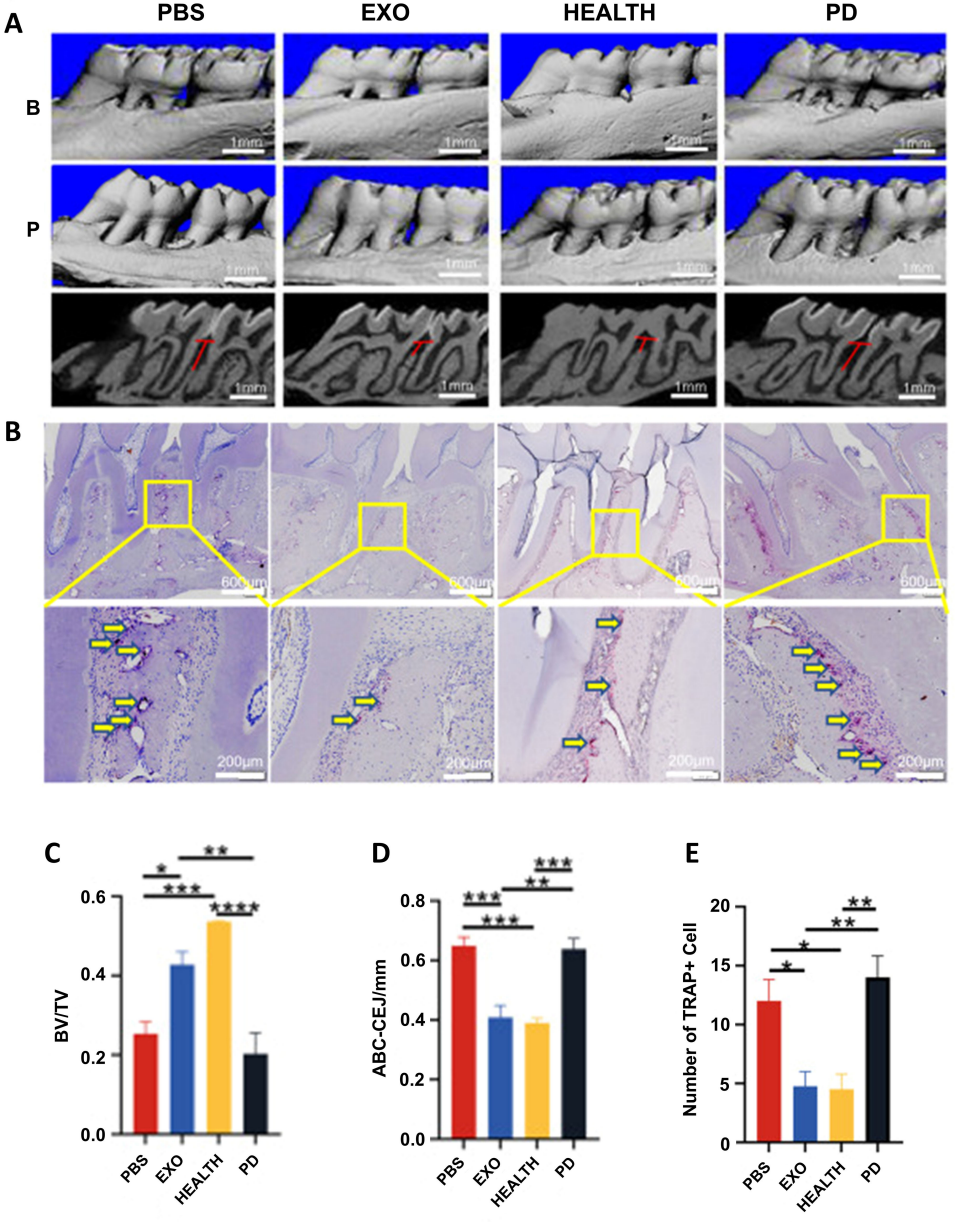
The therapeutic effect of DPSC-EXO on periodontitis in rats. **(A)** Sagittal view of a micro CT scan of the maxillary molars. The red line corresponds to the ABC-CEJ distance. **(B)** The extent of osteoclast infiltration in TRAP-stained sections of periodontal tissue. **(C, D)** BV/TV measurements and distance from ABC to CEJ. **(E)** Quantitative analysis of osteoclasts between the first and second molars *p < 0.05; **p < 0.01; ***p < 0.001; ****p<0.0001. (85). ^©^ 2023 Qiao et al.

Periodontitis is characterized by progressive inflammation and alveolar osteolysis that eventually leads to tooth loss, fundamentally driven by disrupted osteoblast-osteoclast homeostasis (88). Therapeutic strategies must therefore stabilize the immune microenvironment, activate mesenchymal stem cells (bone marrow mesenchymal stem cells, BMSCs/PDLSCs), and enhance osteogenic differentiation (20). Recently, exosomes derived from various types of cells have been reported to inhibit periodontal bone loss and promote tissue regeneration (26, 51). Currently, there is evidence that exosomes can promote the repair of experimental periodontitis in rats through anti-inflammatory as well as modulation of stem cell proliferation and osteogenesis (**Figure 5**) (85).

Specifically, immune cell-derived exosomes can enhance periodontal bone regeneration through targeted molecular delivery. Myeloid dendritic cell exosomes deliver miR-335 to BMSCs to stimulate proliferation and osteogenic differentiation (89), while LPS-stimulated monocyte exosomes upregulate expression of osteogenic genes in MSCs to facilitate bone regeneration (90). Exosomes from different types of macrophages have been shown to have different functional effects on osteogenesis, Macrophage-derived exosomes of types M0 and M2 have all been reported to contribute to osteogenesis (91, 92). M2-exosomes, in particular, induce BMSC differentiation into osteoblasts by targeting salt-inducible kinases 2 and 3 via miRNA-5106 (93) and inhibit osteoclastogenesis by releasing miR-1227-5p (94), delivering CYLD to inactivate STAT3 (95), and regulating the Pyruvate kinase (PK)M2/Hypoxia-inducible factor 1-alpha (HIF-1α) signaling axis (96). Furthermore, reparative M2-exosomes further deliver IL-10 mRNA to activate the IL-10/IL-10R pathway, simultaneously upregulating IL-10 expression in BMSCs and bone marrow-derived macrophage, which drives BMSC osteogenesis and inhibits osteoclast formation in periodontitis mice (20).

Periodontitis-associated bone loss primarily involves the RANKL/RANK pathway (97, 98), and the Wnt and TGF-β signaling pathways further participate in the pathogenesis of periodontitis through their roles in periodontal tissue development, including fibrinogenesis promotion, and osteogenesis regulation (99, 100). Exosomes play pivotal roles in counteracting this destruction, they promote bone formation by activating and enhancing the specific osteogenic differentiation of key mesenchymal stem cells (BMSCs and PDLSCs) through the delivery of specific cargoes (85, 101–105). Li et al. found that vascular endothelial cell-derived exosomes facilitated the osteogenic differentiation and inhibited adipogenic differentiation of BMSCs by inhibiting STAT1 via miR-5p-72106_14 (106). Chen et al. found that miR-184 was down-regulated in the exosome of LPS-pretreated DFSCs, which attenuates oxidative stress, reduces inflammatory responses, and promotes osteogenic differentiation by targeting PPARα inhibition, activating the Akt pathway, and inhibiting the JNK pathway in PDLSCs (107). Likewise, Liu et al. demonstrated that exosomal lncRNA HCP5 promotes osteogenic differentiation of hPDLSCs through the miR-24-3p/HO1/P38/ELK1 pathway, thereby promoting bone regeneration (108). Mechanistically, exosomes inhibit Axin1 expression while upregulating β-catenin (109), and modulate Wnt (110) and RANKL (51) pathways to promote osteoblast generation and suppress osteoclast formation. Moreover, certain exosome subtypes carry surface-bound Wnt and TGF-β molecules that directly activate osteogenic pathways (111, 112), collectively enhancing bone repair and regeneration.

Beyond this direct osteogenic stimulation, exosomes critically ensure the success of bone regeneration by orchestrating a supportive vascular niche (113). They effectively promote angiogenesis both *in vitro* and *in vivo*, even under hyperglycemic conditions, with the extent of neovascularization demonstrating a positive correlation with exosome concentration (114). Spatiotemporal coupling with angiogenesis (i.e., the precise coordination of where and when new blood vessels form) is essential for functional regeneration. An adequate vascular network supports the growth of cells associated with tissue production by delivering nutrient and oxygen, and removing metabolic waste (115). H-vessels, in particular, regulate bone vascularization, recruit osteoblasts and combine osteogenesis with angiogenesis (116). Exosomes establish vascularized bone niches by stimulating endothelial cells (ECs) proliferation/migration and upregulating VEGF/HIF-1α (117, 118). Notably, a self-amplifying circuit between BMSCs and ECs is triggered by EC-exosomes, which upregulate ZBTB16 in BMSCs to promote osteoprogenitor conversion; these osteoprogenitors reciprocally enhance H-type vessel formation via HIF-1α activation (119). Molecularly, exosomes may positively regulate angiogenesis and osteogenesis by mediating SOX2 (120), OTULIN (121) and other factors to regulate the Wnt/β-catenin signaling pathway, while activating the HIF-1α/VEGF and BMP-2/Smad1/RUNX2 signaling pathways (117). Additionally, Behera et al. proposed that the exosomal lnc-H19 uptakes endogenous miR-106 and regulates the expression of the angiogenic factor Angpt1, thereby activating lnc-H19/Tie2-NO signaling in mesenchymal and ECs, exerting angiogenic effects and promoting osteogenesis (115).

In conclusion, bone regeneration and repair is a complex physiological process that involves the synergistic action of many different cell types, such as MSCs, osteoblasts, osteoclasts, vascular endothelial cells and immune cells (122). Exosomes extracted from a variety of the above cells have been reported to promote bone repair by facilitating cell proliferation and osteogenic differentiation, inhibiting osteoclast formation, promoting blood vessel formation, and modulating immunity.

### 4.3 Periodontal ligament regeneration

PDLSCs are considered to be the main functional cells for periodontal repair and regeneration because of their multipotent differentiation capacity (osteogenic, chondrogenic, adipogenic) and immunomodulatory properties, enabling reconstruction of osteoid/PDL-like tissues and alveolar bone. However, under inflammatory conditions, PDLSCs exhibit significantly impaired regenerative potential (123, 124).

*In vivo* studies confirm superior periodontal regeneration in exosome-treated rats, including newly formed bone and PDL formation (26). MSC-exos rescue this PDLSCs dysfunction through synergistic mechanisms: They suppress TNF-α/LPS-induced inflammation in human PDLSCs by inhibiting NF-κB activation—reducing apoptosis and osteogenic impairment while enhancing proliferation/differentiation (125–127)—with GMSC-exos specifically attenuating inflammation via NF-κB/Wnt5a axis modulation (128). Concurrently, exosomal biomolecules engage TLR to regulate immune responses (129–131), and by blocking the TLR4/NF-κB axis (a key driver of periodontitis-associated osteogenic suppression), MSC-exos mitigate inflammatory damage (21). Furthermore, exosomes can influence cellular function by regulating cellular energy metabolism. MSC-exos deliver glycolytic enzymes and other Adenosine triphosphate (ATP)-generating enzymes (e.g., adenylate kinase, nucleoside-diphosphate kinase) that elevate ATP production via anaerobic metabolism (132–134), providing energy for cell survival and activating CD73-mediated adenosine receptor signaling to trigger pro-survival AKT/ERK pathways, thereby enhancing PDLSC migration and proliferation (26). M2-exos promotes aerobic glycolysis-mediated osteogenic differentiation of hPDLSCs by targeting TRIM26-induced PKM ubiquitination through significantly elevated miR-6879-5p (135).

## 5 Engineered exosomes

Exosomes have the capacity to load multiple bioactive components involved in the regulation of cellular communication and function in a paracrine manner. Consequently, their potential is amplified through two primary strategic paradigms: integration with biomaterial scaffolds to control spatiotemporal release, and direct engineering of the vesicles themselves to enhance targeting and therapeutic payload.

Exosomes have been combined with cells and materials, including hydrogels, biomaterials, and nanomaterials, to establish active tissue engineering complexes, which represent a promising future treatment for injured or defective tissues (136). Compared to free exosomes, combining exosomes with tissue-engineered scaffolds were suitable for stable and sustained exosome release, and may be advantageous for osteogenic induction and tissue repair. Moreover, exosomes often co-modify the scaffold surface with other pro-tissue-forming factors and anti-inflammatory factors to repair tissue damage. For example, Ti6Al4V scaffolds functionalized with engineered Smurf1-Exosome from BMSCs activate BMP/Smad signaling pathway in BMSCs and induce macrophage M2 polarization to promote osseointegration (137), while acellular fish scale scaffolds loaded with BMSC-exos enhance BMSC adhesion, proliferation, and osteogenic differentiation *in vitro*, accelerating bone regeneration *in vivo* (138).

**Figure 6:**
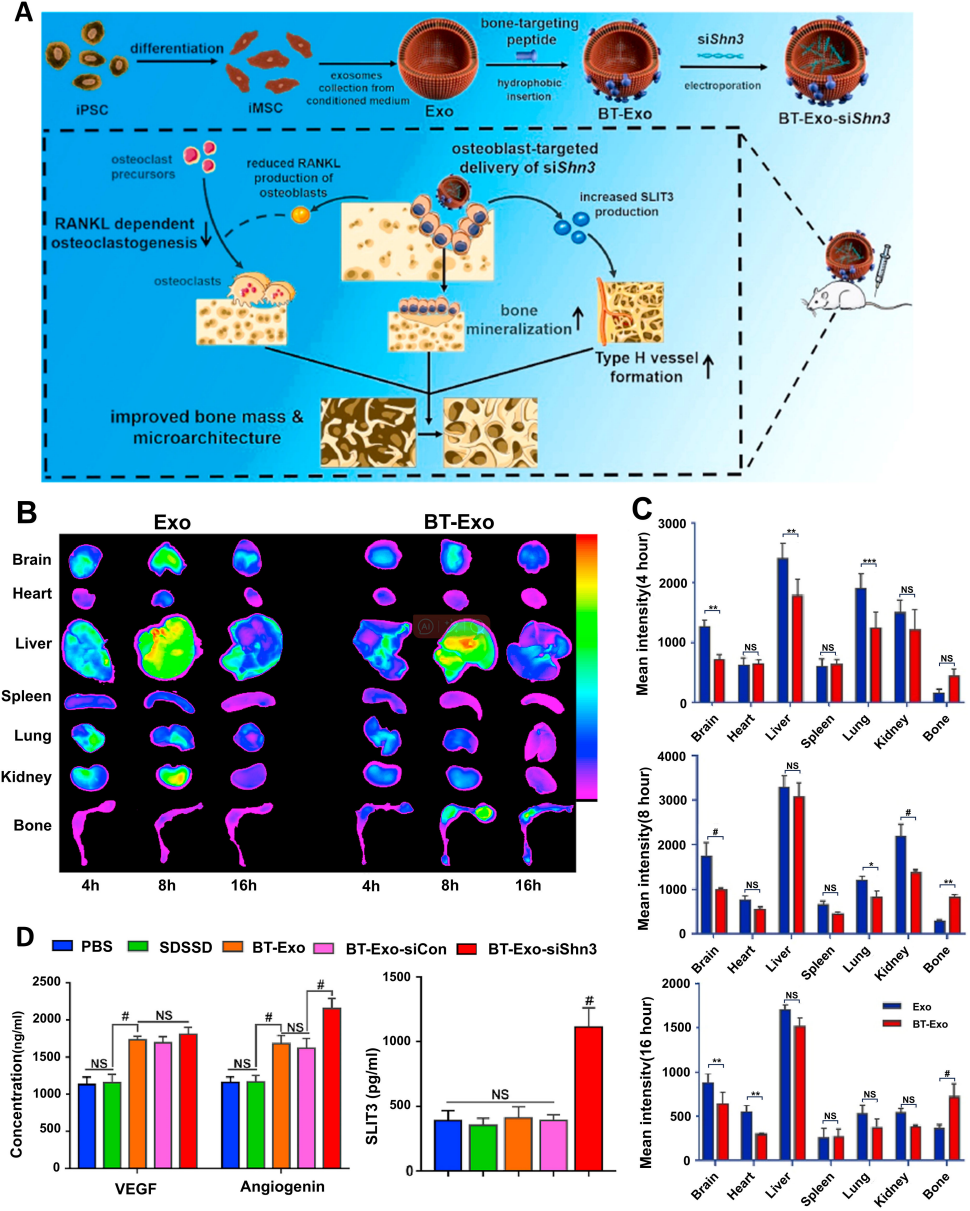
Bt-Exo-siShn3 promotes bone regeneration. **(A)** Preparation of BT-Exo-siShn3 and its therapeutic mechanism. **(B)** Biodistribution of DiR-labeled Exo in mouse. **(C)** Fluorescence intensity of DiR-labeled Exo in different organs. **(D)** Expression levels of VEGF, angiopoietin and SLIT3 in the co-culture system after different treatments. NS, no significant difference; *p < 0.05; **p < 0.01; ***p < 0.001; ^#^p<0.0001. (144). Adapted from Cui et al., 2022, ^©^ 2021 The Authors.

Beyond native vesicles, exosomes function as engineered delivery systems for therapeutic cargo (proteins, nucleic acids, lipids), leveraging their lipid bilayer structure for efficient intracellular transfer alongside lower immunogenicity and superior stability compared to synthetic nanoparticles (139, 140). Distinct from manufactured liposomal carriers, exosomes are endogenously assembled in cells, packaging a complex of biomolecules (e.g., targeting moieties, adhesion proteins) into their lipid bilayer that confers multifunctional properties (141). Since their composition reflects the donor cell’s phenotype, exosomes from specific sources possess inherent functional tendencies; immune cell-derived exosomes, for example, strongly modulate immunity (142). This intrinsic targeting capability makes them ideal for achieving precise drug delivery and enhanced therapeutic outcomes when loaded with therapeutic cargo. At present, there are two primary strategies to load exogenous drugs into exosomes (1): Pre-loading via genetic modification of parent cells to produce inherently functionalized exosomes (2); Post-loading through direct cargo incorporation into isolated exosomes. For example, BMSC-exos loaded with miR-26a via immunomodulatory peptide (DP7-C), activating the mTOR pathway to stimulate osteogenesis and suppress periodontitis (143); exosomes overexpressing miR-181b (Exo-181b) that inhibit PRKCD/AKT signaling to promote M2 macrophage polarization and bone formation (57). Moreover, the application of exosomes is undergoing a paradigm shift from serving as generic carriers to functioning as “precision-guided molecular therapeutics.” This advanced strategy synergistically leverages the inherent homing capabilities of exosomes by integrating exogenous targeting ligands with highly specific cargo (e.g., siRNA, miRNA), thereby achieving multi-faceted therapeutic outcomes. A example is bone-targeting exosome system (Bt-Exo-siShn3), an engineered exosome delivery system, which was constructed from iMSC-derived exosomes loaded with siShn3 via electroporation and conjugated to a bone-targeting peptide. This complex specifically delivers siRNA to osteoblasts, mediating Shn3 gene silencing to enhance osteogenic differentiation, promote H-type vessel formation, and inhibit osteoclastogenesis—collectively facilitating bone regeneration (**Figure 6**) (144).

In summary, engineered exosomes successfully integrate the biological properties of natural vesicles with the precision and controllability of synthetic nanotechnology. While inheriting the inherent advantages of natural exosomes—such as low immunogenicity, high biocompatibility, and excellent biological barrier penetration—they have been further optimized through targeted modifications to effectively reduce off-target effects and enhance therapeutic precision and efficacy. As a result, engineered exosomes demonstrate significant potential in the treatment of periodontitis, offering the prospect of overcoming the limitations of conventional delivery systems and providing a more efficient and safe strategy for disease intervention.

## 6 Clinical challenges and future directions

Exosomes hold considerable promise for the treatment of periodontitis; however, several significant challenges remain, and their translation into clinical practice remains distant.

1. Challenges in large-scale production, storage, and transportation continue to limit the clinical application of exosomes (145). Current exosome yields remain low, and common methods such as ultracentrifugation are time-consuming and costly (146). Furthermore, exosomes require strict storage conditions. Studies have demonstrated that storage at 4°C can lead to aggregation and structural damage, while storage at –80°C—though it preserves particle size and concentration—may compromise certain functional properties of EVs (147). The use of cryoprotectants such as trehalose and the development of lyophilization techniques represent potential strategies to improve storage stability (148).
2. Lack of unified and efficient standards for production, isolation, and purification. Unlike synthetic carriers, exosomes are formed through intracellular assembly. Their molecular composition and biological functions depend not only on the cell type but are also susceptible to variations in culture conditions—such as bioreactor type, culture medium, duration, and cell confluence—which may alter their biological characteristics. Studies indicate that exosomes derived from the same parental cells can still exhibit heterogeneity (149). Moreover, different isolation methods yield exosomes with considerable variability in size, cargo, and function, resulting in poor batch-to-batch consistency and challenges in achieving reproducible production. This complicates comparative studies and treatment standardization (150). Quality control (QC) is essential to ensure the reproducibility of exosomes in research and to verify the purity, homogeneity, and integrity of exosome-based products prior to therapeutic use. Although the International Society for Extracellular Vesicles (ISEV) has issued guidelines recommending QC assessments, the processes for exosome isolation, optimization, and purification still lack universal standardization (151). Therefore, establishing unified protocols spanning isolation, characterization, and production, along with developing stable, controllable, and cost-effective large-scale manufacturing processes, is imperative for clinical translation.
3. Technical immaturity in therapeutic cargo loading and insufficient understanding of surface modification impacts. First, current methods for encapsulating therapeutic molecules—such as nucleic acids and proteins—into exosomes (including physical, chemical, and biological approaches) often compromise the integrity and functionality of the phospholipid membrane and surface proteins. In addition, lipid insertion strategies may introduce cytotoxicity (150, 152, 153). Second, although natural exosomes exhibit low immunogenicity, engineering modifications—particularly the introduction of exogenous proteins or chemical molecules—may alter their immunogenic profile and provoke unintended immune responses.
4. Evidence for exosome therapy in periodontitis remains insufficient. Current research remains largely confined to *in vitro* and animal models, with a notable lack of large-scale clinical trials validating their safety and efficacy. Some studies suggest that long-term use and genetically modified exosomes may pose unknown side effects (154). Moreover, as previously mentioned, the immune microenvironment in periodontitis is highly dynamic, and exosomes play a dual role in this context. Their regulatory networks remain poorly defined, and translating these complex—and at times contradictory—immunomodulatory effects into safe, predictable, and effective clinical treatments for periodontitis remains a major hurdle. Although certain substances within exosomes have been found to exert beneficial effects on immune regulation and tissue regeneration in periodontal inflammation, the composition of exosomal cargo remains highly complex. Within the specific pathological environment of periodontitis, our understanding of the cargo carried by exosomes from different cellular sources and their comprehensive effects on target cells remains limited.

In summary, critical obstacles—including challenges in scalable production, lack of standardized quality control, and immature engineering technologies—hinder the translation of exosome-based therapies into clinical products. From a current perspective, exosome-based treatment for periodontitis remains at a preliminary stage, and the associated costs for translational research and clinical application are expected to be substantial (155).

## 7 Conclusions

Exosomes, secreted by nearly all cell types, function as key mediators of intercellular communication by carrying and delivering bioactive molecules such as proteins and nucleic acids. This enables them to exert the primary functions of their parent cells. Owing to their low immunogenicity, capacity for delivery across biological barriers, and multifaceted regulatory roles, exosomes have emerged as significant vectors for “cell-free therapeutics”. Their applications in disease diagnosis and treatment are consequently receiving increasing attention.

This review systematically elaborates on the therapeutic potential of exosomes in overcoming the limitations of traditional periodontal regeneration strategies. This potential is realized primarily through modulating the inflammatory immune microenvironment and directly promoting the repair of diverse periodontal tissues. Furthermore, engineered modifications of exosomes or their integration with biocompatible scaffolds can significantly enhance their targeting specificity and regenerative efficacy.

Although experimental studies have confirmed the therapeutic effects of exosomes against periodontitis (**Table 1**), their clinical translation faces several challenges. These include low yield, a lack of standardized protocols for isolation and characterization, difficulties in controlling heterogeneity, and inadequate assessment of long-term safety. Therefore, future efforts necessitate exploring large-scale, standardized production technologies and advancing engineering designs to enhance their yield and purity, thereby expanding the frontiers of their application in precision medicine. Additionally, more comprehensive and in-depth investigations are required to elucidate the detailed mechanisms and establish the definitive therapeutic efficacy of exosomes for the diagnosis and treatment of periodontitis.

**Table 1:** Lists the characteristics and application scenarios published over the past 5 years regarding the use of cell-derived exosomes for periodontal regeneration *in vivo*.

Source of exosomes	Engineering and preconditioning	Animal model defect type	Dose	Frequency	Vehicle	Results
M2-macrophages (20)		Ligature-induced periodontitis model in mice	30 µL (50 µg/mL)	every 3 days for 7 days	PBS	Reduce osteoclast infiltration, and alleviate alveolar bone destruction via IL-10 mRNA
neutrophils (39)	induction of apoptosis with staurosporine	Ligature-induced periodontitis model in rats	30 μL	twice weekly for 4 weeks	GelMA@MP196	Antibacterial, inhibit alveolar bone loss, relieve periodontitis
M2 macrophages (48)	Melatonin	Ligature-induced periodontitis model in rat	200 μL	every 2 days for 2 weeks	GelMA hydrogel	Reduce inflammation, inhibit bone resorption
GMSCs (51)	TNF-α	Ligature-induced periodontitis model in mice	20 μg (1 µg/µL)	every 3 days for 7 days	PBS	Reduce osteoclast infiltration, and alleviate alveolar bone destruction
DPSCs (85)		Ligature-induced periodontitis and LPS local injection in mice	10 µL (50 µg/mL)	every other day for 30 days	PBS	Reduce inflammatory factor (TNF-α), decrease M1-type macrophages, thicken the periodontal epithelial fibrous layer, reduce osteoclast infiltration, and alleviate alveolar bone destruction
hBMSCs (108)	sh-NC/sh-HCP5	Ligature-induced periodontitis in mice		every 3 days for 6 weeks	PBS	Exosomal lncRNA HCP5 inhibits pro-inflammatory factor expression and promotes bone regeneration
BMSCs (143)	miR-26a, DP7-C	Ligature-induced periodontitis model in mice	10 μL(50 μg/mL)	every 3 days for 11 days	PBS	Reduce alveolar bone loss
hucMSCs (156)		Ligature-induced periodontitis in mice	50 µL (1.2×10^10^ particles/mL)	twice daily for 8 days	hydrogel	Reduce inflammation, reduce osteoclasts, inhibit bone resorption
Macrophages (157)	DP7-C	Ligature-induced periodontitis in mice	10µL (1.2 µg/µL)	every 3 days for 2 weeks	PBS	Reduce inflammation, reduce osteoclasts, inhibit bone resorption
Schwann cell(SC) (158)		maxillary bone defect (2×2×2 mm³) in rats		Single implantation	GelMA hydrogel	Promote nerve regeneration, angiogenesis, bone regeneration and orderly arrangement of collagen fibers
Stem cells from human exfoliated deciduous teeth (SHED) (159)		Ligature-induced periodontitis model in rats	100 µL(5 µg/µL)	every 2 days for 2 weeks	PBS	Reduce inflammatory factors (TNF-α/IL-1β/IL-6), promote osteogenic gene expression, attenuate periodontal tissue destruction
Human embryonic kidney cells (293T/17) (160)	CXCR4 and miR-126	Ligature-induced periodontitis model in rats	300 μg	Single local injection	PBS	Reduce bone loss, osteoclast activation, and M1 macrophages; Increase M2 polarization
PDLSCs (161)	miR-31-5p mimics/inhibitors NG-PDLSCs/HG-PDLSCs: 5.5 mM/33 mM glucose	Ligature-induced periodontitis model in mice	300 μg	Weekly for 4 weeks	PBS	Reduce alveolar bone loss (NG > HG) and osteoclasts
PDLSCs (162)	miR-205-5p	gingival sulcus injection of LPS (2 mg/ml) every 2 days for 4 weeks in rats	10 μg	every 2 days for 4 weeks	PBS	Reduce inflammatory cell infiltration and TNF-α/IL-1β/IL-6 levels, decrease Th17 cells, and increased Treg cells
Macrophages (163)		LPS local injection model in mice	100 μg (caudal vein injection)	every 2 days for a total of ten times	PBS	M1-exos attenuates Cementoblast mineralization; M2-exos augmentes Cementoblast mineralization
PDLSCs (164)		bilateral periodontal defects (2×2×2 mm³) in rats	2 μg/μL	Single implantation	Gelatin-Sodium Alginate Hydrogel (Gel-Alg)	Promote alveolar bone regeneration
DPSCs (165)		Ligature-induced periodontitis model in mice	5μL (7.5 × 10^8^ particles)	every 2 days for 7 days	physiological saline (PS)	Reduce alveolar bone loss
PDLSCs (166)		buccal alveolar bone defect of the right mandibular first molar (3 × 1.5 × 2 mm³) in rats	150 μg/μL	Single implantation (8 weeks)	Matrigel/β-TCP	Promote alveolar bone regeneration, promote the orderly arrangement of periodontal fibers
GMSCs (167)	TNF-α	Ligature-induced periodontitis model in mice	12.5 μM miR-PEI-NPs	Injected on day 0 and day 3 for 7 days	Polyethylenimine nanoparticles (PEI-NPs)	Reduce alveolar bone loss, reduce ATF6β expression
DPSCs (52)		Ligature-induced periodontitis model in mice	50 μg	Single local injection	Chitosan hydrogel (CS)	Reduce bone loss and epithelial damage, decrease pro-inflammatory cytokines

In conclusion, compared to conventional therapies, exosomes represent a transformative cell-free therapeutic vector, offering a novel paradigm for periodontal treatment and demonstrating immense potential for clinical application.

## References

[1] Eke PI Thornton-Evans G Dye B Genco R . Advances in surveillance of periodontitis: the centers for disease control and prevention periodontal disease surveillance project. J Periodontol. (2012) 83:1337–42. doi: 10.1902/jop.2012.110676, PMID: 22324489 PMC6004792

[2] Kwon T Lamster IB Levin L . Current concepts in the management of periodontitis. Int Dent J. (2021) 71:462–76. doi: 10.1111/idj.12630, PMID: 34839889 PMC9275292

[3] Petersen PE Yamamoto T . Improving the oral health of older people: the approach of the who global oral health programme. Community Dent Oral Epidemiol. (2005) 33:81–92. doi: 10.1111/j.1600-0528.2004.00219.x, PMID: 15725170

[4] Sanz M Marco Del Castillo A Jepsen S Gonzalez-Juanatey JR D’Aiuto F Bouchard P . Periodontitis and cardiovascular diseases: consensus report. J Clin Periodontol. (2020) 47:268–88. doi: 10.1111/jcpe.13189, PMID: 32011025 PMC7027895

[5] Kaur S White S Bartold M . Periodontal disease as a risk factor for rheumatoid arthritis: A systematic review. JBI Libr Syst Rev. (2012) 10:1–12. doi: 10.11124/jbisrir-2012-288, PMID: 27820156

[6] Genco RJ Sanz M . Clinical and public health implications of periodontal and systemic diseases: an overview. Periodontol 2000. (2020) 83:7–13. doi: 10.1111/prd.12344, PMID: 32385880

[7] Gomes-Filho IS Coelho JMF Miranda SS Cruz SS Trindade SC Cerqueira EMM . Severe and moderate periodontitis are associated with acute myocardial infarction. J Periodontol. (2020) 91:1444–52. doi: 10.1002/jper.19-0703, PMID: 32219849

[8] Slots J . Periodontitis: facts, fallacies and the future. Periodontol 2000. (2017) 75:7–23. doi: 10.1111/prd.12221, PMID: 28758294

[9] Yu S Liu J Ma X Wei Z Ye H Zou L . Artesunate carbon dots for attenuating periodontal inflammation and promoting bone regenerationviaactivation of ampk. Colloids Surf B Biointerfaces. (2025) 254:114873. doi: 10.1016/j.colsurfb.2025.114873, PMID: 40499487

[10] Li C Yue X Gao H Meng Q Qu J Chen Y . Injectable composite hydrogels based on minocycline and nir photothermal therapy for antimicrobial and bone regeneration. Biomater Adv. (2025) 177:214397. doi: 10.1016/j.bioadv.2025.214397, PMID: 40644855

[11] Hynes K Menicanin D Gronthos S Bartold PM . Clinical utility of stem cells for periodontal regeneration. Periodontol 2000. (2012) 59:203–27. doi: 10.1111/j.1600-0757.2012.00443.x, PMID: 22507067

[12] Nuñez J Vignoletti F Caffesse RG Sanz M . Cellular therapy in periodontal regeneration. Periodontol 2000. (2019) 79:107–16. doi: 10.1111/prd.12250, PMID: 30892768

[13] Liu J Ruan J Weir MD Ren K Schneider A Wang P . Periodontal bone-ligament-cementum regeneration via scaffolds and stem cells. Cells. (2019) 8:537. doi: 10.3390/cells8060537, PMID: 31167434 PMC6628570

[14] Lin H Chen H Zhao X Chen Z Zhang P Tian Y . Advances in mesenchymal stem cell conditioned medium-mediated periodontal tissue regeneration. J Transl Med. (2021) 19:456. doi: 10.1186/s12967-021-03125-5, PMID: 34736500 PMC8567704

[15] Zheng C Chen J Liu S Jin Y . Stem cell-based bone and dental regeneration: A view of microenvironmental modulation. Int J Oral Sci. (2019) 11:23. doi: 10.1038/s41368-019-0060-3, PMID: 31423011 PMC6802669

[16] Deng J Zhang Y Xie Y Zhang L Tang P . Cell transplantation for spinal cord injury: tumorigenicity of induced pluripotent stem cell-derived neural stem/progenitor cells. Stem Cells Int. (2018) 2018:5653787. doi: 10.1155/2018/5653787, PMID: 29535771 PMC5817265

[17] Dostert G Mesure B Menu P Velot É . How do mesenchymal stem cells influence or are influenced by microenvironment through extracellular vesicles communication? Front Cell Dev Biol. (2017) 5:6. doi: 10.3389/fcell.2017.00006, PMID: 28224125 PMC5293793

[18] Wang X He L Huang X Zhang S Cao W Che F . Recent progress of exosomes in multiple myeloma: pathogenesis, diagnosis, prognosis and therapeutic strategies. Cancers (Basel). (2021) 13:1635. doi: 10.3390/cancers13071635, PMID: 33915822 PMC8037106

[19] Raposo G Stoorvogel W . Extracellular vesicles: exosomes, microvesicles, and friends. J Cell Biol. (2013) 200:373–83. doi: 10.1083/jcb.201211138, PMID: 23420871 PMC3575529

[20] Chen X Wan Z Yang L Song S Fu Z Tang K . Exosomes derived from reparative M2-like macrophages prevent bone loss in murine periodontitis models via il-10 mrna. J Nanobiotechnology. (2022) 20:110. doi: 10.1186/s12951-022-01314-y, PMID: 35248085 PMC8898524

[21] Liu J Chen T Lei P Tang X Huang P . Exosomes released by bone marrow mesenchymal stem cells attenuate lung injury induced by intestinal ischemia reperfusion via the tlr4/nf-κb pathway. Int J Med Sci. (2019) 16:1238–44. doi: 10.7150/ijms.35369, PMID: 31588189 PMC6775266

[22] Chen W Huang Y Han J Yu L Li Y Lu Z . Immunomodulatory effects of mesenchymal stromal cells-derived exosome. Immunol Res. (2016) 64:831–40. doi: 10.1007/s12026-016-8798-6, PMID: 27115513

[23] He C Zheng S Luo Y Wang B . Exosome theranostics: biology and translational medicine. Theranostics. (2018) 8:237–55. doi: 10.7150/thno.21945, PMID: 29290805 PMC5743472

[24] Lin H Chen H Zhao X Ding T Wang Y Chen Z . Advances of exosomes in periodontitis treatment. J Transl Med. (2022) 20:279. doi: 10.1186/s12967-022-03487-4, PMID: 35729576 PMC9210629

[25] Wang R Ji Q Meng C Liu H Fan C Lipkind S . Role of gingival mesenchymal stem cell exosomes in macrophage polarization under inflammatory conditions. Int Immunopharmacol. (2020) 81:106030. doi: 10.1016/j.intimp.2019.106030, PMID: 31796385

[26] Chew JRJ Chuah SJ Teo KYW Zhang S Lai RC Fu JH . Mesenchymal stem cell exosomes enhance periodontal ligament cell functions and promote periodontal regeneration. Acta Biomater. (2019) 89:252–64. doi: 10.1016/j.actbio.2019.03.021, PMID: 30878447

[27] Garbern JC Lee RT . Cardiac stem cell therapy and the promise of heart regeneration. Cell Stem Cell. (2013) 12:689–98. doi: 10.1016/j.stem.2013.05.008, PMID: 23746978 PMC3756309

[28] Yan XZ Yang F Jansen JA De Vries RB Van Den Beucken JJ . Cell-based approaches in periodontal regeneration: A systematic review and meta-analysis of periodontal defect models in animal experimental work. Tissue Eng Part B Rev. (2015) 21:411–26. doi: 10.1089/ten.TEB.2015.0049, PMID: 25929285

[29] Liang X Ding Y Zhang Y Tse HF Lian Q . Paracrine mechanisms of mesenchymal stem cell-based therapy: current status and perspectives. Cell Transplant. (2014) 23:1045–59. doi: 10.3727/096368913x667709, PMID: 23676629

[30] Williams DW Greenwell-Wild T Brenchley L Dutzan N Overmiller A Sawaya AP . Human oral mucosa cell atlas reveals a stromal-neutrophil axis regulating tissue immunity. Cell. (2021) 184:4090–104.e15. doi: 10.1016/j.cell.2021.05.013, PMID: 34129837 PMC8359928

[31] Cohn ZA Hirsch JG . The influence of phagocytosis on the intracellular distribution of granule-associated components of polymorphonuclear leucocytes. J Exp Med. (1960) 112:1015–22. doi: 10.1084/jem.112.6.1015, PMID: 13694489 PMC2137323

[32] Kim TS Silva LM Theofilou VI Greenwell-Wild T Li L Williams DW . Neutrophil extracellular traps and extracellular histones potentiate il-17 inflammation in periodontitis. J Exp Med. (2023) 220:e20221751. doi: 10.1084/jem.20221751, PMID: 37261457 PMC10236943

[33] Mousa AO Al Hussaini AHA Hussein HM . The potential role of reactive oxygen species produced by low-density neutrophils in periodontitis. Eur J Dent. (2024) 18:1142–8. doi: 10.1055/s-0044-1782211, PMID: 38744332 PMC11479733

[34] Shan Y Zhong J Sun Q Gao W Zhang C Chen H . Dual nanozymes-loaded core-shell microneedle patches with antibacterial and nets-degradation bifunctional properties for periodontitis treatment. Bioact Mater. (2025) 53:161–77. doi: 10.1016/j.bioactmat.2025.07.003, PMID: 40688015 PMC12274864

[35] Vitkov L Klappacher M Hannig M Krautgartner WD . Extracellular neutrophil traps in periodontitis. J Periodontal Res. (2009) 44:664–72. doi: 10.1111/j.1600-0765.2008.01175.x, PMID: 19453857

[36] Kaspi H Semo J Abramov N Dekel C Lindborg S Kern R . Msc-ntf (Nurown^®^) exosomes: A novel therapeutic modality in the mouse lps-induced ards model. Stem Cell Res Ther. (2021) 12:72. doi: 10.1186/s13287-021-02143-w, PMID: 33468250 PMC7814377

[37] Chen L Liu Y Wang Z Zhang L Xu Y Li Y . Mesenchymal stem cell-derived extracellular vesicles protect against abdominal aortic aneurysm formation by inhibiting net-induced ferroptosis. Exp Mol Med. (2023) 55:939–51. doi: 10.1038/s12276-023-00986-2, PMID: 37121969 PMC10238484

[38] Morishima Y Kawabori M Yamazaki K Takamiya S Yamaguchi S Nakahara Y . Intravenous administration of mesenchymal stem cell-derived exosome alleviates spinal cord injury by regulating neutrophil extracellular trap formation through exosomal mir-125a-3p. Int J Mol Sci. (2024) 25:2406. doi: 10.3390/ijms25042406, PMID: 38397083 PMC10889446

[39] Zhuo H Zhang S Wang H Deng J Zhang X . Gelatin methacryloyl @Mp196/exos hydrogel induced neutrophil apoptosis and macrophage M2 polarization to inhibit periodontal bone loss. Colloids Surf B Biointerfaces. (2025) 248:114466. doi: 10.1016/j.colsurfb.2024.114466, PMID: 39729702

[40] Yang J Xie Y Xia Z Ji S Yang X Yue D . Hucmsc-exo induced N2 polarization of neutrophils: implications for angiogenesis and tissue restoration in wound healing. Int J Nanomedicine. (2024) 19:3555–75. doi: 10.2147/ijn.S458295, PMID: 38638364 PMC11024985

[41] Taghavi-Farahabadi M Mahmoudi M Rezaei N Hashemi SM . Wharton’s jelly mesenchymal stem cells exosomes and conditioned media increased neutrophil lifespan and phagocytosis capacity. Immunol Invest. (2021) 50:1042–57. doi: 10.1080/08820139.2020.1801720, PMID: 32777963

[42] Mahmoudi M Taghavi-Farahabadi M Rezaei N Hashemi SM . Comparison of the effects of adipose tissue mesenchymal stromal cell-derived exosomes with conditioned media on neutrophil function and apoptosis. Int Immunopharmacol. (2019) 74:105689. doi: 10.1016/j.intimp.2019.105689, PMID: 31207404

[43] Zhou S Zhu Y Wu Y Zhang X Kong X Zhao X . New insights on metabolic reprogramming in macrophage plasticity. Int Immunopharmacol. (2025) 157:114797. doi: 10.1016/j.intimp.2025.114797, PMID: 40339492

[44] Wynn TA Vannella KM . Macrophages in tissue repair, regeneration, and fibrosis. Immunity. (2016) 44:450–62. doi: 10.1016/j.immuni.2016.02.015, PMID: 26982353 PMC4794754

[45] Gordon S Martinez FO . Alternative activation of macrophages: mechanism and functions. Immunity. (2010) 32:593–604. doi: 10.1016/j.immuni.2010.05.007, PMID: 20510870

[46] Sun X Gao J Meng X Lu X Zhang L Chen R . Polarized macrophages in periodontitis: characteristics, function, and molecular signaling. Front Immunol. (2021) 12:763334. doi: 10.3389/fimmu.2021.763334, PMID: 34950140 PMC8688840

[47] Yang J Zhu Y Duan D Wang P Xin Y Bai L . Enhanced activity of macrophage M1/M2 phenotypes in periodontitis. Arch Oral Biol. (2018) 96:234–42. doi: 10.1016/j.archoralbio.2017.03.006, PMID: 28351517

[48] Cui Y Hong S Xia Y Li X He X Hu X . Melatonin engineering M2 macrophage-derived exosomes mediate endoplasmic reticulum stress and immune reprogramming for periodontitis therapy. Adv Sci (Weinh). (2023) 10:e2302029. doi: 10.1002/advs.202302029, PMID: 37452425 PMC10520618

[49] Yue C Cao J Wong A Kim JH Alam S Luong G . Human bone marrow stromal cell exosomes ameliorate periodontitis. J Dent Res. (2022) 101:1110–8. doi: 10.1177/00220345221084975, PMID: 35356822 PMC9305845

[50] Hu P Liang L Li B Xia W . Heterocoagulation between coal and quartz particles studied by the mineral heterocoagulation quantifying system. Minerals Eng. (2019) 138:7–13. doi: 10.1016/j.mineng.2019.04.029

[51] Nakao Y Fukuda T Zhang Q Sanui T Shinjo T Kou X . Exosomes from tnf-α-treated human gingiva-derived mscs enhance M2 macrophage polarization and inhibit periodontal bone loss. Acta Biomater. (2021) 122:306–24. doi: 10.1016/j.actbio.2020.12.046, PMID: 33359765 PMC7897289

[52] Shen Z Kuang S Zhang Y Yang M Qin W Shi X . Chitosan hydrogel incorporated with dental pulp stem cell-derived exosomes alleviates periodontitis in mice via a macrophage-dependent mechanism. Bioactive Materials. (2020) 5:1113–26. doi: 10.1016/j.bioactmat.2020.07.002, PMID: 32743122 PMC7371600

[53] Lo Sicco C Reverberi D Balbi C Ulivi V Principi E Pascucci L . Mesenchymal stem cell-derived extracellular vesicles as mediators of anti-inflammatory effects: endorsement of macrophage polarization. Stem Cells Transl Med. (2017) 6:1018–28. doi: 10.1002/sctm.16-0363, PMID: 28186708 PMC5442783

[54] Chen Y Dong J Li J Li J Lu Y Dong W . Engineered macrophage-derived exosomes via click chemistry for the treatment of osteomyelitis. J Mater Chem B. (2024) 12:10593–604. doi: 10.1039/d4tb01346h, PMID: 39315933

[55] Shen D He Z . Mesenchymal stem cell-derived exosomes regulate the polarization and inflammatory response of macrophages via mir-21-5p to promote repair after myocardial reperfusion injury. Ann Transl Med. (2021) 9:1323. doi: 10.21037/atm-21-3557, PMID: 34532460 PMC8422151

[56] Zhao J Li X Hu J Chen F Qiao S Sun X . Mesenchymal stromal cell-derived exosomes attenuate myocardial ischaemia-reperfusion injury through mir-182-regulated macrophage polarization. Cardiovasc Res. (2019) 115:1205–16. doi: 10.1093/cvr/cvz040, PMID: 30753344 PMC6529919

[57] Liu W Yu M Chen F Wang L Ye C Chen Q . A novel delivery nanobiotechnology: engineered mir-181b exosomes improved osteointegration by regulating macrophage polarization. J Nanobiotechnology. (2021) 19:269. doi: 10.1186/s12951-021-01015-y, PMID: 34493305 PMC8424816

[58] Li R Li D Wang H Chen K Wang S Xu J . Exosomes from adipose-derived stem cells regulate M1/M2 macrophage phenotypic polarization to promote bone healing via mir-451a/mif. Stem Cell Res Ther. (2022) 13:149. doi: 10.1186/s13287-022-02823-1, PMID: 35395782 PMC8994256

[59] Wang Z Maruyama K Sakisaka Y Suzuki S Tada H Suto M . Cyclic stretch force induces periodontal ligament cells to secrete exosomes that suppress il-1β Production through the inhibition of the nf-κb signaling pathway in macrophages. Front Immunol. (2019) 10:1310. doi: 10.3389/fimmu.2019.01310, PMID: 31281309 PMC6595474

[60] Yuan X Cao H Wang J Tang K Li B Zhao Y . Immunomodulatory effects of calcium and strontium co-doped titanium oxides on osteogenesis. Front Immunol. (2017) 8:1196. doi: 10.3389/fimmu.2017.01196, PMID: 29033930 PMC5626827

[61] Wu H Li L Ma Y Chen Y Zhao J Lu Y . Regulation of selective pparγ Modulators in the differentiation of osteoclasts. J Cell Biochem. (2013) 114:1969–77. doi: 10.1002/jcb.24534, PMID: 23494891

[62] Yamaguchi T Movila A Kataoka S Wisitrasameewong W Ruiz Torruella M Murakoshi M . Proinflammatory M1 macrophages inhibit rankl-induced osteoclastogenesis. Infect Immun. (2016) 84:2802–12. doi: 10.1128/iai.00461-16, PMID: 27456834 PMC5038061

[63] Yang J Park OJ Kim J Kwon Y Yun CH Han SH . Modulation of macrophage subtypes by irf5 determines osteoclastogenic potential. J Cell Physiol. (2019) 234:23033–42. doi: 10.1002/jcp.28863, PMID: 31127629

[64] Bai X Wang Y Ma X Yang Y Deng C Sun M . Periodontal ligament cells-derived exosomes promote osteoclast differentiation via modulating macrophage polarization. Sci Rep. (2024) 14:1465. doi: 10.1038/s41598-024-52073-9, PMID: 38233593 PMC10794214

[65] Wang L Wang J Jin Y Gao H Lin X . Oral administration of all-trans retinoic acid suppresses experimental periodontitis by modulating the th17/treg imbalance. J Periodontol. (2014) 85:740–50. doi: 10.1902/jop.2013.130132, PMID: 23952076

[66] Zheng Y Dong C Yang J Jin Y Zheng W Zhou Q . Exosomal microrna-155-5p from pdlscs regulated th17/treg balance by targeting sirtuin-1 in chronic periodontitis. J Cell Physiol. (2019) 234:20662–74. doi: 10.1002/jcp.28671, PMID: 31016751

[67] Proto JD Doran AC Gusarova G Yurdagul A Jr. Sozen E Subramanian M . Regulatory T cells promote macrophage efferocytosis during inflammation resolution. Immunity. (2018) 49:666–77.e6. doi: 10.1016/j.immuni.2018.07.015, PMID: 30291029 PMC6192849

[68] Li B Zheng SG . How regulatory T cells sense and adapt to inflammation. Cell Mol Immunol. (2015) 12:519–20. doi: 10.1038/cmi.2015.65, PMID: 26277895 PMC4579659

[69] Wang R Liang Q Zhang Q Zhao S Lin Y Liu B . Ccl2-induced regulatory T cells balance inflammation through macrophage polarization during liver reconstitution. Adv Sci (Weinh). (2024) 11:e2403849. doi: 10.1002/advs.202403849, PMID: 39352304 PMC11615773

[70] Garlet GP Cardoso CR Mariano FS Claudino M De Assis GF Campanelli AP . Regulatory T cells attenuate experimental periodontitis progression in mice. J Clin Periodontol. (2010) 37:591–600. doi: 10.1111/j.1600-051X.2010.01586.x, PMID: 20642629

[71] Zhang Y Guo J Jia R . Treg: A promising immunotherapeutic target in oral diseases. Front Immunol. (2021) 12:667862. doi: 10.3389/fimmu.2021.667862, PMID: 34177907 PMC8222692

[72] Kadowaki N . Dendritic cells: A conductor of T cell differentiation. Allergol Int. (2007) 56:193–9. doi: 10.2332/allergolint.R-07-146, PMID: 17646736

[73] Elashiry M Elashiry MM Elsayed R Rajendran M Auersvald C Zeitoun R . Dendritic cell derived exosomes loaded with immunoregulatory cargo reprogram local immune responses and inhibit degenerative bone disease *in vivo*. J Extracell Vesicles. (2020) 9:1795362. doi: 10.1080/20013078.2020.1795362, PMID: 32944183 PMC7480413

[74] El-Awady AR Elashiry M Morandini AC Meghil MM Cutler CW . Dendritic cells a critical link to alveolar bone loss and systemic disease risk in periodontitis: immunotherapeutic implications. Periodontol 2000. (2022) 89:41–50. doi: 10.1111/prd.12428, PMID: 35244951 PMC9018591

[75] Aiello S Rocchetta F Longaretti L Faravelli S Todeschini M Cassis L . Extracellular vesicles derived from T regulatory cells suppress T cell proliferation and prolong allograft survival. Sci Rep. (2017) 7:11518. doi: 10.1038/s41598-017-08617-3, PMID: 28912528 PMC5599553

[76] Okoye IS Coomes SM Pelly VS Czieso S Papayannopoulos V Tolmachova T . Microrna-containing T-regulatory-cell-derived exosomes suppress pathogenic T helper 1 cells. Immunity. (2014) 41:89–103. doi: 10.1016/j.immuni.2014.05.019, PMID: 25035954 PMC4104030

[77] Zhang Y Chen J Fu H Kuang S He F Zhang M . Exosomes derived from 3d-cultured mscs improve therapeutic effects in periodontitis and experimental colitis and restore the th17 cell/treg balance in inflamed periodontium. Int J Oral Sci. (2021) 13:43. doi: 10.1038/s41368-021-00150-4, PMID: 34907166 PMC8671433

[78] Lin Z Xiong Y Meng W Hu Y Chen L Chen L . Exosomal pd-L1 induces osteogenic differentiation and promotes fracture healing by acting as an immunosuppressant. Bioact Mater. (2022) 13:300–11. doi: 10.1016/j.bioactmat.2021.10.042, PMID: 35224310 PMC8844834

[79] Rivera A Siracusa MC Yap GS Gause WC . Innate cell communication kick-starts pathogen-specific immunity. Nat Immunol. (2016) 17:356–63. doi: 10.1038/ni.3375, PMID: 27002843 PMC4949486

[80] Bosshardt DD . The periodontal pocket: pathogenesis, histopathology and consequences. Periodontol 2000. (2018) 76:43–50. doi: 10.1111/prd.12153, PMID: 29194796

[81] Lv J Liu Y Jia S Zhang Y Tian H Li J . Carbon monoxide-releasing molecule-3 suppresses tumor necrosis factor-α- and interleukin-1β-induced expression of junctional molecules on human gingival fibroblasts via the heme oxygenase-1 pathway. Mediators Inflammation. (2020) 2020:6302391. doi: 10.1155/2020/6302391, PMID: 32410860 PMC7204158

[82] Galenko-Yaroshevsky PA Slavinskiy AА Todorov SS Popkov VL Shelemekh OV Lebedeva SA . The effect of zinc complex of N-isopropenylimidazole on the morphological characteristics of gum tissues in experimental endodontic-periodontal lesions in rats. Res Results Pharmacol. (2023) 9:1–12. doi: 10.18413/rrpharmacology.9.10040

[83] Tiwari S Mahale SA Walvekar AK Patil RD Harish S Mehta A . Efficacy of colloidal nanosilver tooth gel in the management of orodental conditions: A prospective, randomized, triple arm, parallel, double-blind controlled interventional clinical study. J Oral Health Community Dentistry. (2023) 17:49–56. doi: 10.5005/jp-journals-10062-0166

[84] Takeuchi H Nakamura E Yamaga S Amano A . Porphyromonas gingivalis infection induces lipopolysaccharide and peptidoglycan penetration through gingival epithelium. Front Oral Health. (2022) 3:845002. doi: 10.3389/froh.2022.845002, PMID: 35211692 PMC8861192

[85] Qiao X Tang J Dou L Yang S Sun Y Mao H . Dental pulp stem cell-derived exosomes regulate anti-inflammatory and osteogenesis in periodontal ligament stem cells and promote the repair of experimental periodontitis in rats. Int J Nanomedicine. (2023) 18:4683–703. doi: 10.2147/ijn.S420967, PMID: 37608819 PMC10441659

[86] Zheng Y Dong X Wang X Wang J Chen S He Y . Exosomes derived from adipose tissue-derived mesenchymal stromal cells prevent medication-related osteonecrosis of the jaw through il-1ra. Int J Mol Sci. (2023) 24:8694. doi: 10.3390/ijms24108694, PMID: 37240036 PMC10218172

[87] Kou X Xu X Chen C Sanmillan ML Cai T Zhou Y . The fas/fap-1/cav-1 complex regulates il-1ra secretion in mesenchymal stem cells to accelerate wound healing. Sci Transl Med. (2018) 10:eaai8524. doi: 10.1126/scitranslmed.aai8524, PMID: 29540618 PMC6310133

[88] Zhou R Shen L Yang C Wang L Guo H Yang P . Periodontitis may restrain the mandibular bone healing via disturbing osteogenic and osteoclastic balance. Inflammation. (2018) 41:972–83. doi: 10.1007/s10753-018-0751-5, PMID: 29460020

[89] Cao Z Wu Y Yu L Zou L Yang L Lin S . Exosomal mir-335 derived from mature dendritic cells enhanced mesenchymal stem cell-mediated bone regeneration of bone defects in athymic rats. Mol Med. (2021) 27:20. doi: 10.1186/s10020-021-00268-5, PMID: 33637046 PMC7913386

[90] Ekström K Omar O Granéli C Wang X Vazirisani F Thomsen P . Monocyte exosomes stimulate the osteogenic gene expression of mesenchymal stem cells. PloS One. (2013) 8:e75227. doi: 10.1371/journal.pone.0075227, PMID: 24058665 PMC3776724

[91] Kang M Huang CC Lu Y Shirazi S Gajendrareddy P Ravindran S . Bone regeneration is mediated by macrophage extracellular vesicles. Bone. (2020) 141:115627. doi: 10.1016/j.bone.2020.115627, PMID: 32891867 PMC8107826

[92] Xia Y He XT Xu XY Tian BM An Y Chen FM . Exosomes derived from M0, M1 and M2 macrophages exert distinct influences on the proliferation and differentiation of mesenchymal stem cells. PeerJ. (2020) 8:e8970. doi: 10.7717/peerj.8970, PMID: 32355576 PMC7185029

[93] Xiong Y Chen L Yan C Zhou W Yu T Sun Y . M2 macrophagy-derived exosomal mirna-5106 induces bone mesenchymal stem cells towards osteoblastic fate by targeting salt-inducible kinase 2 and 3. J Nanobiotechnology. (2020) 18:66. doi: 10.1186/s12951-020-00622-5, PMID: 32345321 PMC7189726

[94] Chen S Liu J Zhu L . M2-like macrophage-derived exosomes inhibit osteoclastogenesis via releasing mir-1227-5p. Immunobiology. (2025) 230:152861. doi: 10.1016/j.imbio.2024.152861, PMID: 39700638

[95] Guo ZY Yin NN Li XF Wang MM Sui XN Jiang CD . Exosomes secreted from M2-polarized macrophages inhibit osteoclast differentiation via cyld. Tissue Cell. (2025) 93:102645. doi: 10.1016/j.tice.2024.102645, PMID: 39671756

[96] Zhang Y Liang Y Zhou Y . M2 polarization of raw264.7-derived exosomes inhibits osteoclast differentiation and inflammation via pkm2/hif-1α Axis. Immunol Invest. (2025) 54:1–15. doi: 10.1080/08820139.2025.2525896, PMID: 40583280

[97] Tsukasaki M Komatsu N Nagashima K Nitta T Pluemsakunthai W Shukunami C . Host defense against oral microbiota by bone-damaging T cells. Nat Commun. (2018) 9:701. doi: 10.1038/s41467-018-03147-6, PMID: 29453398 PMC5816021

[98] Hajishengallis G Chavakis T Lambris JD . Current understanding of periodontal disease pathogenesis and targets for host-modulation therapy. Periodontol 2000. (2020) 84:14–34. doi: 10.1111/prd.12331, PMID: 32844416 PMC7457922

[99] Kuznetsova AV Popova OP Danilova TI Latyshev AV Yanushevich OO Ivanov AA . Effects of ecm components on periodontal ligament stem cell differentiation under conditions of disruption of wnt and tgf-β Signaling pathways. J Funct Biomater. (2025) 16:94. doi: 10.3390/jfb16030094, PMID: 40137373 PMC11942902

[100] Wei X Liu Q Guo S Wu Y . Role of wnt5a in periodontal tissue development, maintenance, and periodontitis: implications for periodontal regeneration (Review). Mol Med Rep. (2021) 23:167. doi: 10.3892/mmr.2020.11806, PMID: 33398377 PMC7821221

[101] Narayanan R Huang CC Ravindran S . Hijacking the cellular mail: exosome mediated differentiation of mesenchymal stem cells. Stem Cells Int. (2016) 2016:3808674. doi: 10.1155/2016/3808674, PMID: 26880957 PMC4736778

[102] Gao J Wu Z . M2 macrophage-derived exosomes enable osteogenic differentiation and inhibit inflammation in human periodontal ligament stem cells through promotion of cxcl12 expression. BMC Oral Health. (2024) 24:1070. doi: 10.1186/s12903-024-04831-4, PMID: 39261847 PMC11391719

[103] Deng Y Xiao J Huang X Cao Z . Macrophage-derived exosomes rescue the tnf-α-suppressed osteo-/cementogenic differentiation of hpdlcs. Oral Dis. (2024) 30:5232–42. doi: 10.1111/odi.14947, PMID: 38566464

[104] Wang M Li J Ye Y Chen D Song J . Shed-derived exosomes improve the repair capacity and osteogenesis potential of hpdlcs. Oral Dis. (2023) 29:1692–705. doi: 10.1111/odi.14153, PMID: 35152542

[105] Liu Y Zeng L Wang W Yang Y Wang Z Liu J . Human bone marrow mesenchymal stem cell exosome-derived mir-335-5p promotes osteogenic differentiation of human periodontal ligament stem cells to alleviate periodontitis by downregulating dkk1. Nan Fang Yi Ke Da Xue Xue Bao. (2023) 43:420–7. doi: 10.12122/j.issn.1673-4254.2023.03.12, PMID: 37087587 PMC10122733

[106] Li H Wang J Xie X Chen Y Zheng Q He J . Exosome-derived mir-5p-72106_14 in vascular endothelial cells regulates fate determination of bmscs. Toxicol Appl Pharmacol. (2024) 482:116793. doi: 10.1016/j.taap.2023.116793, PMID: 38123076

[107] Chen L Zhang J Yu J Guo S Tian W . Lps pretreated dental follicle stem cell derived exosomes promote periodontal tissue regeneration via mir-184 and pparα-akt-jnk signaling pathway. Stem Cell Res Ther. (2025) 16:347. doi: 10.1186/s13287-025-04462-8, PMID: 40605003 PMC12224499

[108] Liu Y Zhu J Wang WH Zeng L Yang YL Wang Z . Exosomal lncrna hcp5 derived from human bone marrow mesenchymal stem cells improves chronic periodontitis by mir-24-3p/ho1/P38/elk1 pathway. Heliyon. (2024) 10:e34203. doi: 10.1016/j.heliyon.2024.e34203, PMID: 39104492 PMC11298838

[109] Cui Y Luan J Li H Zhou X Han J . Exosomes derived from mineralizing osteoblasts promote st2 cell osteogenic differentiation by alteration of microrna expression. FEBS Lett. (2016) 590:185–92. doi: 10.1002/1873-3468.12024, PMID: 26763102

[110] Zhang D Xiao W Liu C Wang Z Liu Y Yu Y . Exosomes derived from adipose stem cells enhance bone fracture healing via the activation of the wnt3a/β-catenin signaling pathway in rats with type 2 diabetes mellitus. Int J Mol Sci. (2023) 24:4852. doi: 10.3390/ijms24054852, PMID: 36902283 PMC10003369

[111] Gross JC Chaudhary V Bartscherer K Boutros M . Active wnt proteins are secreted on exosomes. Nat Cell Biol. (2012) 14:1036–45. doi: 10.1038/ncb2574, PMID: 22983114

[112] Webber J Steadman R Mason MD Tabi Z Clayton A . Cancer exosomes trigger fibroblast to myofibroblast differentiation. Cancer Res. (2010) 70:9621–30. doi: 10.1158/0008-5472.Can-10-1722, PMID: 21098712

[113] Sunartvanichkul T Arayapisit T Sangkhamanee SS Chaweewannakorn C Iwasaki K Klaihmon P . Stem cell-derived exosomes from human exfoliated deciduous teeth promote angiogenesis in hyperglycemic-induced human umbilical vein endothelial cells. J Appl Oral Sci. (2023) 31:e20220427. doi: 10.1590/1678-7757-2022-0427, PMID: 37042872 PMC10118382

[114] Qi X Zhang J Yuan H Xu Z Li Q Niu X . Exosomes secreted by human-induced pluripotent stem cell-derived mesenchymal stem cells repair critical-sized bone defects through enhanced angiogenesis and osteogenesis in osteoporotic rats. Int J Biol Sci. (2016) 12:836–49. doi: 10.7150/ijbs.14809, PMID: 27313497 PMC4910602

[115] Behera J Kumar A Voor MJ Tyagi N . Exosomal lncrna-H19 promotes osteogenesis and angiogenesis through mediating angpt1/tie2-no signaling in cbs-heterozygous mice. Theranostics. (2021) 11:7715–34. doi: 10.7150/thno.58410, PMID: 34335960 PMC8315071

[116] Kusumbe AP Ramasamy SK Adams RH . Coupling of angiogenesis and osteogenesis by a specific vessel subtype in bone. Nature. (2014) 507:323–8. doi: 10.1038/nature13145, PMID: 24646994 PMC4943525

[117] Zhang L Jiao G Ren S Zhang X Li C Wu W . Exosomes from bone marrow mesenchymal stem cells enhance fracture healing through the promotion of osteogenesis and angiogenesis in a rat model of nonunion. Stem Cell Res Ther. (2020) 11:38. doi: 10.1186/s13287-020-1562-9, PMID: 31992369 PMC6986095

[118] Zhang Y Hao Z Wang P Xia Y Wu J Xia D . Exosomes from human umbilical cord mesenchymal stem cells enhance fracture healing through hif-1α-mediated promotion of angiogenesis in a rat model of stabilized fracture. Cell Prolif. (2019) 52:e12570. doi: 10.1111/cpr.12570, PMID: 30663158 PMC6496165

[119] Liu L Zhou N Fu S Wang L Liu Y Fu C . Endothelial cell-derived exosomes trigger a positive feedback loop in osteogenesis-angiogenesis coupling via up-regulating zinc finger and btb domain containing 16 in bone marrow mesenchymal stem cell. J Nanobiotechnology. (2024) 22:721. doi: 10.1186/s12951-024-03002-5, PMID: 39563357 PMC11577908

[120] Wu F Song C Zhen G Jin Q Li W Liang X . Exosomes derived from bmscs in osteogenic differentiation promote type H blood vessel angiogenesis through mir-150-5p mediated metabolic reprogramming of endothelial cells. Cell Mol Life Sci. (2024) 81:344. doi: 10.1007/s00018-024-05371-4, PMID: 39133273 PMC11335269

[121] Luo Z Peng W Xu Y Xie Y Liu Y Lu H . Exosomal otulin from M2 macrophages promotes the recovery of spinal cord injuries via stimulating wnt/β-catenin pathway-mediated vascular regeneration. Acta Biomater. (2021) 136:519–32. doi: 10.1016/j.actbio.2021.09.026, PMID: 34551329

[122] Ho-Shui-Ling A Bolander J Rustom LE Johnson AW Luyten FP Picart C . Bone regeneration strategies: engineered scaffolds, bioactive molecules and stem cells current stage and future perspectives. Biomaterials. (2018) 180:143–62. doi: 10.1016/j.biomaterials.2018.07.017, PMID: 30036727 PMC6710094

[123] Ikeda H Sumita Y Ikeda M Ikeda H Okumura T Sakai E . Engineering bone formation from human dental pulp- and periodontal ligament-derived cells. Ann BioMed Eng. (2011) 39:26–34. doi: 10.1007/s10439-010-0115-2, PMID: 20614244

[124] Lee JS Yi JK An SY Heo JS . Increased osteogenic differentiation of periodontal ligament stem cells on polydopamine film occurs via activation of integrin and pi3k signaling pathways. Cell Physiol Biochem. (2014) 34:1824–34. doi: 10.1159/000366381, PMID: 25502639

[125] Zhang W Jia L Zhao B Xiong Y Wang YN Liang J . Quercetin reverses tnf−α Induced osteogenic damage to human periodontal ligament stem cells by suppressing the nf−κb/nlrp3 inflammasome pathway. Int J Mol Med. (2021) 47:39. doi: 10.3892/ijmm.2021.4872, PMID: 33537804 PMC7891819

[126] Guo L Sun H Pu J . Gnai3 mediated by lin28a regulates lipopolysaccharide-induced inflammation and osteogenic differentiation in periodontal stem cells by mediating the nf-κb/nlrp3 inflammasome pathway. Arch Oral Biol. (2024) 163:105974. doi: 10.1016/j.archoralbio.2024.105974, PMID: 38636252

[127] Jiang J Zhang N Song H Yang Y Li J Hu X . Oridonin alleviates the inhibitory effect of lipopolysaccharide on the proliferation and osteogenic potential of periodontal ligament stem cells by inhibiting endoplasmic reticulum stress and nf-κb/nlrp3 inflammasome signaling. BMC Oral Health. (2023) 23:137. doi: 10.1186/s12903-023-02827-0, PMID: 36894905 PMC9999511

[128] Sun J Wang Z Liu P Hu Y Li T Yang J . Exosomes derived from human gingival mesenchymal stem cells attenuate the inflammatory response in periodontal ligament stem cells. Front Chem. (2022) 10:863364. doi: 10.3389/fchem.2022.863364, PMID: 35464198 PMC9019468

[129] Liu J Jiang M Deng S Lu J Huang H Zhang Y . Mir-93-5p-containing exosomes treatment attenuates acute myocardial infarction-induced myocardial damage. Mol Ther Nucleic Acids. (2018) 11:103–15. doi: 10.1016/j.omtn.2018.01.010, PMID: 29858047 PMC5852413

[130] Kojima M Gimenes-Junior JA Chan TW Eliceiri BP Baird A Costantini TW . Exosomes in postshock mesenteric lymph are key mediators of acute lung injury triggering the macrophage activation via toll-like receptor 4. FASEB J. (2018) 32:97–110. doi: 10.1096/fj.201700488R, PMID: 28855278

[131] Seo W Eun HS Kim SY Yi HS Lee YS Park SH . Exosome-mediated activation of toll-like receptor 3 in stellate cells stimulates interleukin-17 production by Γδ T cells in liver fibrosis. Hepatology. (2016) 64:616–31. doi: 10.1002/hep.28644, PMID: 27178735

[132] Lai RC Yeo RWY Tan SS Zhang B Yin Y Sze NSK . Mesenchymal stem cell exosomes: the future msc-based therapy? In: Chase LG Vemuri MC , editors. Mesenchymal stem cell therapy. Humana Press, Totowa, NJ (2013). p. 39–61.

[133] Lai RC Yeo RW Tan KH Lim SK . Mesenchymal stem cell exosome ameliorates reperfusion injury through proteomic complementation. Regener Med. (2013) 8:197–209. doi: 10.2217/rme.13.4, PMID: 23477399

[134] Lai RC Yeo RWY Lim SK . Mesenchymal stem cell exosomes. Semin Cell Dev Biol. (2015) 40:82–8. doi: 10.1016/j.semcdb.2015.03.001, PMID: 25765629

[135] Liao X Yang Z Li Y Cui Y Ma L Liang C . M2 macrophage-derived exosome facilitates aerobic glycolysis and osteogenic differentiation of hpdlscs by regulating trim26-induced pkm ubiquitination. Free Radic Biol Med. (2025) 237:88–100. doi: 10.1016/j.freeradbiomed.2025.05.425, PMID: 40449810

[136] Kim HD Amirthalingam S Kim SL Lee SS Rangasamy J Hwang NS . Biomimetic materials and fabrication approaches for bone tissue engineering. Adv Healthc Mater. (2017) 6. doi: 10.1002/adhm.201700612, PMID: 29171714

[137] Xu H Chai Q Xu X Li Z Bao W Man Z . Exosome-functionalized ti6al4v scaffolds promoting osseointegration by modulating endogenous osteogenesis and osteoimmunity. ACS Appl Mater Interfaces. (2022) 14:46161–75. doi: 10.1021/acsami.2c11102, PMID: 36203406

[138] Wang Y Kong B Chen X Liu R Zhao Y Gu Z . Bmsc exosome-enriched acellular fish scale scaffolds promote bone regeneration. J Nanobiotechnology. (2022) 20:444. doi: 10.1186/s12951-022-01646-9, PMID: 36224596 PMC9555002

[139] Lou P Liu S Wang Y Lv K Zhou X Li L . Neonatal-tissue-derived extracellular vesicle therapy (Next): A potent strategy for precision regenerative medicine. Adv Mater. (2023) 35:e2300602. doi: 10.1002/adma.202300602, PMID: 37148469

[140] Ferguson SW Nguyen J . Exosomes as therapeutics: the implications of molecular composition and exosomal heterogeneity. J Control Release. (2016) 228:179–90. doi: 10.1016/j.jconrel.2016.02.037, PMID: 26941033

[141] Mathivanan S Ji H Simpson RJ . Exosomes: extracellular organelles important in intercellular communication. J Proteomics. (2010) 73:1907–20. doi: 10.1016/j.jprot.2010.06.006, PMID: 20601276

[142] Sun D Zhuang X Xiang X Liu Y Zhang S Liu C . A novel nanoparticle drug delivery system: the anti-inflammatory activity of curcumin is enhanced when encapsulated in exosomes. Mol Ther. (2010) 18:1606–14. doi: 10.1038/mt.2010.105, PMID: 20571541 PMC2956928

[143] Lai S Deng L Liu C Li X Fan L Zhu Y . Bone marrow mesenchymal stem cell-derived exosomes loaded with mir-26a through the novel immunomodulatory peptide dp7-C can promote osteogenesis. Biotechnol Lett. (2023) 45:905–19. doi: 10.1007/s10529-023-03376-w, PMID: 37195490

[144] Cui Y Guo Y Kong L Shi J Liu P Li R . A bone-targeted engineered exosome platform delivering sirna to treat osteoporosis. Bioact Mater. (2022) 10:207–21. doi: 10.1016/j.bioactmat.2021.09.015, PMID: 34901540 PMC8636739

[145] Ye Y Zhang X Xie F Xu B Xie P Yang T . An engineered exosome for delivering sgrna: cas9 ribonucleoprotein complex and genome editing in recipient cells. Biomater Sci. (2020) 8:2966–76. doi: 10.1039/d0bm00427h, PMID: 32342086

[146] Schwarz G Ren X Xie W Guo H Jiang Y Zhang J . Engineered exosomes: A promising drug delivery platform with therapeutic potential. Front Mol Biosci. (2025) 12:1583992. doi: 10.3389/fmolb.2025.1583992, PMID: 40417062 PMC12098103

[147] Lőrincz ÁM Timár CI Marosvári KA Veres DS Otrokocsi L Kittel Á . Effect of storage on physical and functional properties of extracellular vesicles derived from neutrophilic granulocytes. J Extracell Vesicles. (2014) 3:25465. doi: 10.3402/jev.v3.25465, PMID: 25536933 PMC4275651

[148] Frank J Richter M de Rossi C Lehr CM Fuhrmann K Fuhrmann G . Extracellular vesicles protect glucuronidase model enzymes during freeze-drying. Sci Rep. (2018) 8:12377. doi: 10.1038/s41598-018-30786-y, PMID: 30120298 PMC6098026

[149] Li S Xu J Qian J Gao X . Engineering extracellular vesicles for cancer therapy: recent advances and challenges in clinical translation. Biomater Sci. (2020) 8:6978–91. doi: 10.1039/d0bm01385d, PMID: 33155579

[150] Chen H Wang L Zeng X Schwarz H Nanda HS Peng X . Exosomes, a new star for targeted delivery. Front Cell Dev Biol. (2021) 9:751079. doi: 10.3389/fcell.2021.751079, PMID: 34692704 PMC8531489

[151] Witwer KW Buzás EI Bemis LT Bora A Lässer C Lötvall J . Standardization of sample collection, isolation and analysis methods in extracellular vesicle research. J Extracell Vesicles. (2013) 2:2. doi: 10.3402/jev.v2i0.20360, PMID: 24009894 PMC3760646

[152] Kim MS Haney MJ Zhao Y Yuan D Deygen I Klyachko NL . Engineering macrophage-derived exosomes for targeted paclitaxel delivery to pulmonary metastases: *in vitro* and *in vivo* evaluations. Nanomedicine. (2018) 14:195–204. doi: 10.1016/j.nano.2017.09.011, PMID: 28982587

[153] Li L Hu S Chen X . Non-viral delivery systems for crispr/cas9-based genome editing: challenges and opportunities. Biomaterials. (2018) 171:207–18. doi: 10.1016/j.biomaterials.2018.04.031, PMID: 29704747 PMC5944364

[154] Zhang J Zhang Y Khanal S Cao D Zhao J Dang X . Synthetic grna/cas9 ribonucleoprotein targeting hbv DNA inhibits viral replication. J Med Virol. (2023) 95:e28952. doi: 10.1002/jmv.28952, PMID: 37455550 PMC10977344

[155] Li YJ Wu JY Liu J Xu W Qiu X Huang S . Artificial exosomes for translational nanomedicine. J Nanobiotechnology. (2021) 19:242. doi: 10.1186/s12951-021-00986-2, PMID: 34384440 PMC8359033

[156] Li K Gu X Zhu Y Guan N Wang J Wang L . Human umbilical cord mesenchymal stem cells-derived exosomes attenuates experimental periodontitis in mice partly by delivering mirnas. Int J Nanomedicine. (2025) 20:2879–99. doi: 10.2147/ijn.S502192, PMID: 40078652 PMC11900796

[157] Lai S Tang N Guo J Deng L Yuan L Zeng L . Immunomodulatory peptide dp7-C mediates macrophage-derived exosomal mir-21b to promote bone regeneration via the socs1/jak2/stat3 axis. Colloids Surf B Biointerfaces. (2025) 253:114709. doi: 10.1016/j.colsurfb.2025.114709, PMID: 40286607

[158] Cui Y Li X He X Zhou X Wang X Lin K . Schwann cell-derived exosomes accelerate periodontal bone regeneration with osteogenesis, angiogenesis, and neurogenesis. J Mater Chem B. (2025) 13:4020–9. doi: 10.1039/d4tb02601b, PMID: 40040598

[159] Yu T Mi N Song Y Xie W . Exosomes mir-92a-3p from human exfoliated deciduous teeth inhibits periodontitis progression via the klf4/pi3k/akt pathway. J Periodontal Res. (2024) 59:771–82. doi: 10.1111/jre.13262, PMID: 38616305

[160] Luo H Chen D Li R Li R Teng Y Cao Y . Genetically engineered cxcr4-modified exosomes for delivery of mir-126 mimics to macrophages alleviate periodontitis. J Nanobiotechnology. (2023) 21:116. doi: 10.1186/s12951-023-01863-w, PMID: 36991451 PMC10061745

[161] Lu J Yu N Liu Q Xie Y Zhen L . Periodontal ligament stem cell exosomes key to regulate periodontal regeneration by mir-31-5p in mice model. Int J Nanomedicine. (2023) 18:5327–42. doi: 10.2147/ijn.S409664, PMID: 37746047 PMC10516219

[162] Kang L Miao Y Jin Y Shen S Lin X . Exosomal mir-205-5p derived from periodontal ligament stem cells attenuates the inflammation of chronic periodontitis via targeting xbp1. Immun Inflammation Dis. (2023) 11:e743. doi: 10.1002/iid3.743, PMID: 36705422 PMC9761342

[163] Zhao Y Huang Y Liu H Tan K Wang R Jia L . Macrophages with different polarization phenotypes influence cementoblast mineralization through exosomes. Stem Cells Int. (2022) 2022:4185972. doi: 10.1155/2022/4185972, PMID: 36159746 PMC9507802

[164] Zhao Y Gong Y Liu X He J Zheng B Liu Y . The experimental study of periodontal ligament stem cells derived exosomes with hydrogel accelerating bone regeneration on alveolar bone defect. Pharmaceutics. (2022) 14:2189. doi: 10.3390/pharmaceutics14102189, PMID: 36297624 PMC9611133

[165] Shimizu Y Takeda-Kawaguchi T Kuroda I Hotta Y Kawasaki H Hariyama T . Exosomes from dental pulp cells attenuate bone loss in mouse experimental periodontitis. J Periodontal Res. (2022) 57:162–72. doi: 10.1111/jre.12949, PMID: 34826339

[166] Lei F Li M Lin T Zhou H Wang F Su X . Treatment of inflammatory bone loss in periodontitis by stem cell-derived exosomes. Acta Biomater. (2022) 141:333–43. doi: 10.1016/j.actbio.2021.12.035, PMID: 34979326

[167] Hayashi C Fukuda T Kawakami K Toyoda M Nakao Y Watanabe Y . Mir-1260b inhibits periodontal bone loss by targeting atf6β Mediated regulation of er stress. Front Cell Dev Biol. (2022) 10:1061216. doi: 10.3389/fcell.2022.1061216, PMID: 36531939 PMC9748617

